# Optoacoustic‐Guided Magnetic Microrobot Platform for Precision Drug Delivery

**DOI:** 10.1002/adma.202511870

**Published:** 2025-10-25

**Authors:** Fan Wang, Erdost Yildiz, Xosé Luís Deán‐Ben, Yan Yu, Daniil Nozdriukhin, Wenbin Kang, Shuaizhong Zhang, Jelena Zinnanti, Devin Sheehan, Ren Hao Soon, Shuxin Lyu, Daniel Razansky, Metin Sitti

**Affiliations:** ^1^ Physical Intelligence Department Max Planck Institute for Intelligent Systems 70569 Stuttgart Germany; ^2^ Institute for Biomedical Engineering Department of Information Technology and Electrical Engineering ETH Zürich Zürich 8093 Switzerland; ^3^ Institute of Pharmacology and Toxicology and Institute for Biomedical Engineering Faculty of Medicine University of Zurich Zurich 8057 Switzerland; ^4^ Department of Mechanical Engineering City University of Hong Kong Hong Kong 999077 China; ^5^ Department of Mechanical and Aerospace Engineering The Hong Kong University of Science and Technology Hong Kong 999077 China; ^6^ School of Mechanical Engineering Yanshan University Qinhuangdao 066004 China; ^7^ School of Medicine and College of Engineering Koç University Istanbul 34450 Turkey

**Keywords:** drug delivery, magnetic nanoparticles, metal–organic frameworks, microrobotics, optoacoustic imaging

## Abstract

Precision drug delivery remains a significant challenge due to limitations in drug loading, targeted release, precise navigation, and real‐time monitoring. Here, the study reports a magnetic microrobot platform (MMP) that integrates high‐capacity drug loading, magnetically actuated collective navigation, controlled drug release, and real‐time 3D optoacoustic imaging in a single system. The MMP exploits synergistic advantages by embedding hard‐magnetic FePt nanoparticles in a degradable ZIF‐8 shell, achieving a drug loading efficiency of ≈93.9% and enabling precise release in response to pH changes and radiofrequency‐induced heating. Reconfigurable swarm behavior strategies significantly enhance the navigation efficiency of microrobots against physiological blood flows within complex cerebral vasculature. The ex vivo and in vivo experiments further demonstrate strong contrast characteristics of the microrobots, enabling high‐resolution visualization of deep vascular structures and dynamic tracking of MMP with real‐time 3D optoacoustic imaging. This multifunctional strategy paves the way for clinical translation and precision therapy in complex biological settings.

## Graphical abstract

Optoacoustically guided FePt@ZIF‐8‐based microrobots are designed with high drug loading capacity, controllable locomotion, and deep‐tissue imaging capability. By integrating navigation, drug release, and real‐time monitoring into a single platform, they provide a versatile tool for precise intervention, opening new possibilities for treating neurological disorders and tumors located in hard‐to‐reach regions.

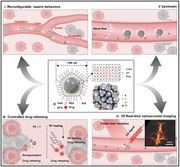

## 1 Introduction

Precision drug delivery remains a critical challenge in modern medicine. Conventional drug delivery systems suffer from significant limitations in key metrics such as drug loading, anatomical targeting specificity, controlled and sustained release, and real‐time monitoring, often leading to suboptimal therapeutic outcomes or iatrogenic complications.^[^ ^1^, ^2^, ^3^ ^]^ In recent years, rapid advances in nanomedicine and microrobotics have stimulated the development of innovative strategies to overcome these limitations.^[^ ^4^ ^]^ Among these, magnetically controlled microrobots have attracted considerable attention for their potential to enable remote navigation in complex physiological environments.^[^ ^5^ ^]^ However, the navigational performance of individual microrobots in high‐speed blood flow and intricate vascular networks is often inadequate, limiting their clinical applicability.^[^ ^6^ ^]^ Consequently, exploiting collective locomotion strategies to harness the synergistic advantages of multiple microrobots has emerged as a promising approach to improve both the precision and efficiency of drug delivery.^[^ ^7^ ^]^ The integration of high drug loading, precisely controlled release, targeted delivery, and real‐time imaging into a single delivery platform remains a formidable challenge.^[^ ^8^ ^]^

Liposomes, metal–organic frameworks (MOFs), and polymeric nanoparticles have long been favored for microrobotics‐combined drug delivery applications due to their excellent biocompatibility.^[^ ^9^, ^10^ ^]^ However, they typically cannot navigate complex blood flows and achieve precise, site‐specific drug release. Similarly, simple magnetic nanoparticles utilize noninvasive, tissue‐penetrating magnetic fields for targeting, but are limited by inefficient drug‐loading capacities and potential cytotoxicity.^[^ ^11^ ^]^ Although previous studies have shown that these magnetic nanoparticles can be functionalized to load drugs via surface charge interactions or chemical modification, such methods often fall short of delivering therapeutic doses and are prone to drug leakage.^[^ ^12^, ^13^ ^]^ As a result, there remains an urgent need for an integrated system that synergistically combines the strengths of multi‐functional nanoparticle types to improve the efficiency and precision of drug delivery.^[^ ^4^ ^]^

Real‐time imaging is also becoming essential for monitoring drug delivery processes. In particular, optoacoustic imaging represents a novel hybrid modality that uses nanosecond laser pulses to induce instantaneous photothermal effects in tissue, thereby converting absorbed optical energy into ultrasound signals.^[^ ^14^, ^15^ ^]^ This technique synergistically combines the rich optical contrast with the high spatial resolution of ultrasound in deep tissues, unaffected by photon scattering.^[^ ^16^ ^]^ Typically, multispectral optoacoustic tomography (MSOT) systems operate in the near‐infrared spectral range between 700 and 1300 nm; a range that not only provides robust excitation of endogenous molecules, which includes hemoglobin, lipids, collagen, melanin, and water, and spectrally distinct exogenous contrast but also allows imaging depths of several centimeters while maintaining spatial resolution of approximately 100–200 µm.^[^ ^17^ ^]^ These capabilities enable real‐time, high‐resolution visualization of deep tissue structures, complex vascular networks, blood oxygenation, and extrinsically administered contrast agents.^[^ ^18^ ^]^ Consequently, researchers can dynamically track the movement of microrobots in vivo, ensuring that therapeutic agents are delivered accurately and efficiently to targeted sites.

In this study, we aimed to build a holistic microrobotic system with integration of optoacoustic imaging with untethered magnetic control of the microrobots into an intravascular drug delivery platform. We investigate a magnetic microrobot platform (MMP) that integrates high drug loading, targeted delivery, precise controlled release, and real‐time imaging into a versatile microrobotic system. The MMP consists of several magnetically programmed nanoparticles (mPNs) with hard magnetic FePt nanoparticles embedded in the core for active propulsion and steering, and an outer layer of zeolitic imidazolate framework 8 (ZIF‐8) that enables high‐capacity drug loading. In addition to them, FePt is one of the few biocompatible hard magnetic compounds in the literature.^[^ ^19^, ^20^ ^]^ For this purpose, we demonstrate the collective navigation of MMP through an in vitro complex cerebral circulation model using external magnetic fields and its drug delivery capabilities and biocompatibility in in vitro models, and track the microbot swarms in the animal model. MMP achieves high drug loading efficiency (up to ≈93.9%) and controlled in vitro drug release triggered by both pH variations and radiofrequency (RF) heating. In addition, real‐time 3D MSOT imaging of circulating MMP is validated in ex vivo porcine heart and brain vessel phantoms as well as in vivo in the mouse vasculature under realistic blood background conditions. Notably, tracking of MMP trajectories allows the reconstruction of high‐resolution 3D murine brain vasculature even without exogenous contrast agents. In short, this study presents a FePt–ZIF‐8 core–shell magnetic microrobot platform with high‐capacity drug loading, magnetically actuated and reconfigurable swarm navigation (chain, oscillation, vortex, dispersion) for efficient blood–brain barrier crossing, and high‐contrast multimodal imaging enabling deep vascular visualization and real‐time 3D tracking. Therefore, the proposed magnetic microrobotic approach holds great promise for transforming precision medicine by enabling real‐time monitoring of on‐demand drug delivery and magnetic actuation within complex vascular environments.

## 2 Results

### 2.1 Design and Actuation of the Magnetic Microrobot Platform

**Figure 1:**
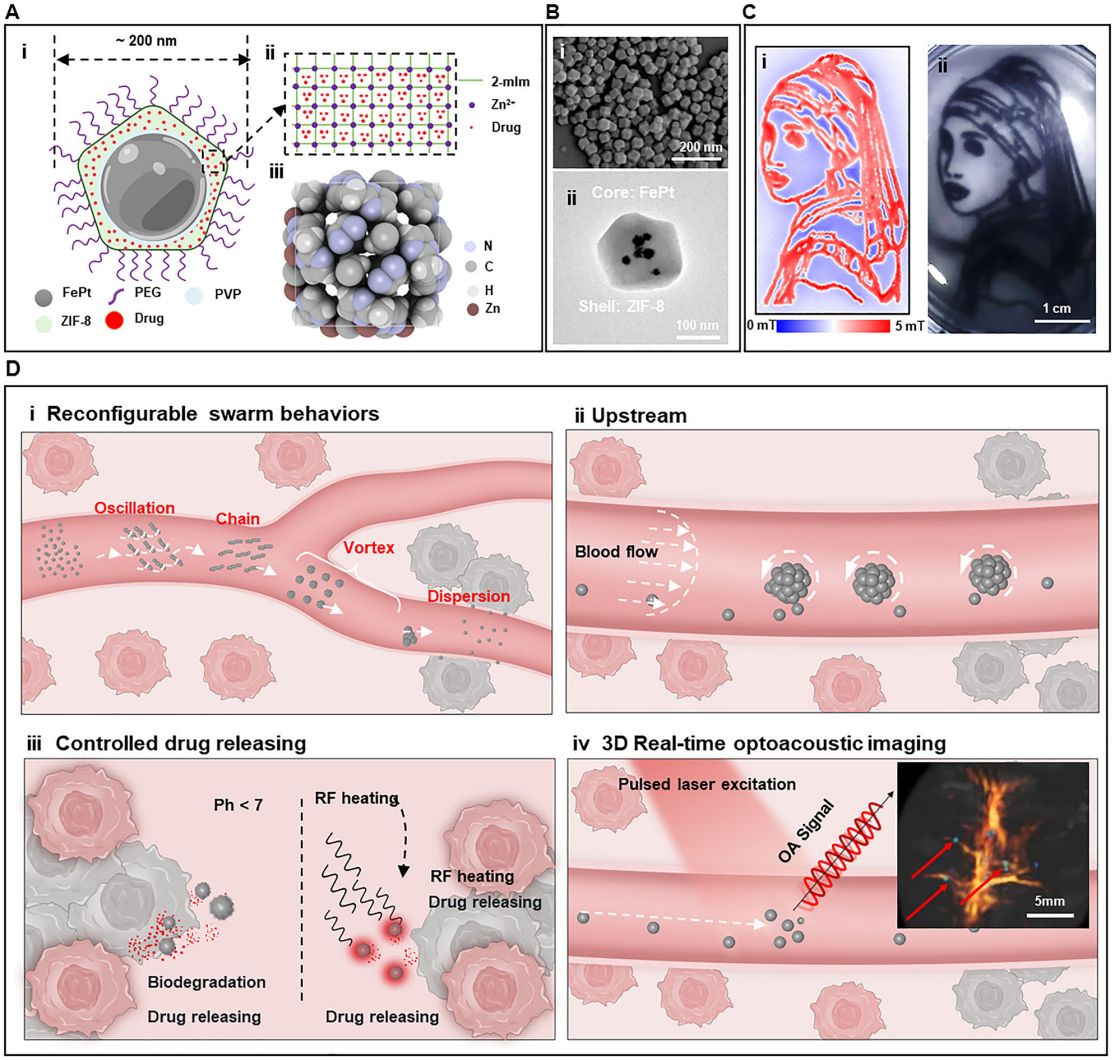
Schematic illustrations of a multifunctional magnetic microrobot platform (MMP) for targeted drug delivery applications. A) (i) Representation of a single engineered nanoparticle with a core‐shell structure, featuring a hard magnetic FePt core and drug‐loaded shell layer. (ii) Drugs are loaded into the shell (ZIF‐8), (iii) with a spacious cavity in the center of the 3D structure of ZIF‐8 for drug encapsulation. B) Scanning electron microscope (SEM) images of mPNs and the transmission electron microscope (TEM) image of a single mPN (scale bar: 200 nm (i) and 100 nm (ii)). C) (i) A weak magnetic field with the Girl with a Pearl Earring pattern. (ii) MMP demonstrates rapid responsiveness to small patterned, attractive magnetic field articles, exemplified by their interaction with the famous painting of “Girl with a Pearl Earring” pattern magnetic field. (scale bar: 1 cm). D) Illustration depicting the MMP (i) reconfigurable swarm behaviors, (ii) upstream movement, (iii) controlled drug delivery by PH and RF, and 3D real‐time imaging with multi‐spectral optoacoustic tomography (MSOT).

We propose an MMP composed of several magnetically programmed nanoparticles. Every single mPN contains mainly hard magnetic FePt nanoparticles in its core, which enables propulsion and steering, and is coated with ZIF‐8 as its shell to achieve high‐capacity drug loading (**Figure** **1**A(i), Notes S1 and S2, Supporting Information). Poly(ethylene glycol) (PEG) coatings on mPNs shield the surface from opsonization and phagocytosis, thereby prolonging systemic circulation time.^[^ ^21^, ^22^ ^]^ As shown in Figure 1A(ii,iii), ZIF‐8 consists of zinc ions and imidazolate ligands, has inherent porous properties, high drug loading capacity, and undergoes pH‐sensitive degradation. This design ensures controlled drug release after reaching a targeted site in the body. The scanning electron microscope (SEM) images of mPNs and the transmission electron microscope (TEM) image of a single mPN (Figure 1B(i,ii)) indicate that the average nanoparticle size is approximately 200 ± 15 nm, with a core‐shell structure consisting of multiple FePt nanoparticles as the core and ZIF‐8 as the shell. In addition, these mPNs exhibit excellent magnetic controllability, allowing them to respond rapidly to weak patterned magnetic fields (Figure 1C(i,ii)). This magnetic actuation capability enhances their precision and efficiency in drug delivery applications.

Based on the design of mPNs, this MMP integrates high drug loading capacity, intelligent magnetic actuation, controlled drug release, and real‐time 3D imaging into a single system (Figure 1D). First, the MMP must be able to be actively navigated and magnetically controlled in the highly dynamic blood flow environment. Intelligent swarm behaviors overcome the limitations associated with a single mPN, including limited drug loading and the risk of loss during manipulation, and are critical for effective drug delivery (Figure 1D(i)). Flexible reconfigurable swarm behaviors (oscillation, chain, vortex, and dispersion) allow the MMP to navigate within complex vascular environments. In addition, to efficiently access the circulatory system, the mPNs can travel against the vascular blood flow, allowing them to effectively reach deep areas (Figure 1D(ii)).^[^ ^23^ ^]^ In addition, MMP, designed for drug delivery, should possess mechanisms for controlled drug release (Figure 1D(iii)). One of the most commonly used approaches is to exploit the pH gradient in the body, where environments with a pH less than 7, indicative of acidic conditions in pathological tissues, such as brain tumors, trigger drug release.^[^ ^24^ ^]^ This pH‐dependent release mechanism can be enhanced by external RF heating, which accelerates the drug release process and ensures timely and targeted delivery to diseased regions.^[^ ^25^ ^]^ Real‐time tracking of these particle swarms is accomplished using advanced MSOT imaging, allowing researchers to monitor their physiological location and trajectory and ensure their accurate positioning (Figure 1D(iv)). By collecting and analyzing time‐lapse image data, the trajectory of the MMP can be tracked in real time to enable the reconstruction of high‐resolution 3D vasculature.

### 2.2 Synthesis and Characterization of the MMP

**Figure 2:**
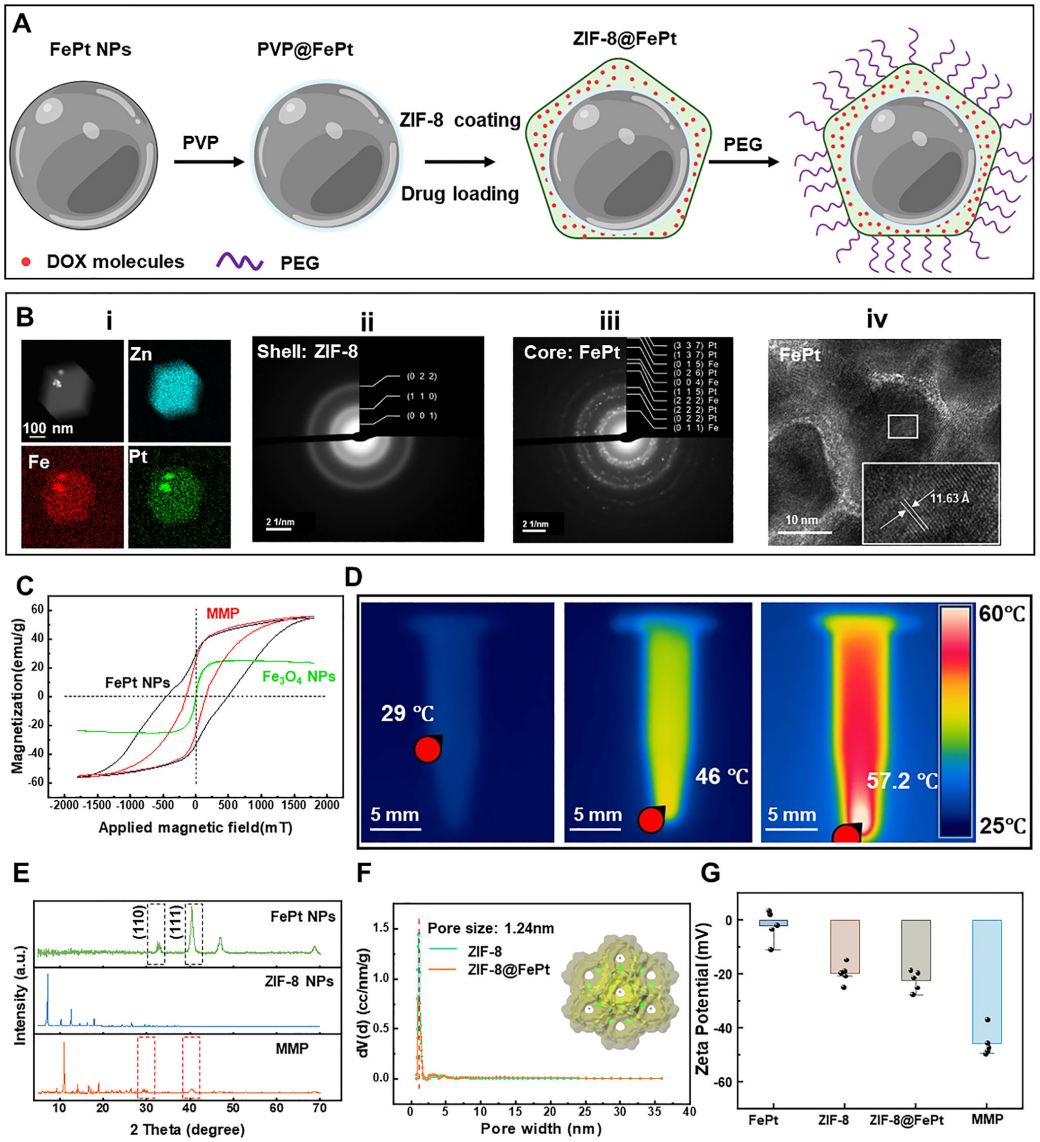
Characterization of the MMP. A) Schematic of the synthetic route of the mPN of the MMP. B) (i) STEM image of a single ZIF‐8@FePt nanoparticle without drugs and the Zn, Fe, Pt mapping. (ii) and (iii) the SAED patterns of the shell of ZIF‐8 and the core of FePt. (iv) HRTEM image of a representative FePt core and its enlarged image. C) Hysteresis loop measurements for dry FePt, MMP, and Fe_3_O_4_ nanoparticles, with magnetic field increments of 0.5 mT. Curves have been normalized by the maximum magnetic moment obtained at 1800 mT.  D) Infrared thermal imaging of MMP dispersed in PBS during RF heating. E) XRD test results of FePt nanoparticles, ZIF‐8 nanoparticles, and MMP. F) BET analysis of the pore size distribution for ZIF‐8 and ZIF‐8@FePt nanoparticles. G) The Zeta potential of FePt nanoparticles, ZIF‐8 nanoparticles, ZIF‐8@FePt nanoparticles, and MMP in PBS solution, and the concentration of all is 100 µg mL^−1^.

We develop the MMP composed of core‐shell structured mPNs composed of PEG/ZIF‐8@FePt to construct biocompatible magnetic microrobots. The mPNs are prepared via a multistep chemical synthesis process (**Figure** **2**A). The modification process with polyvinylpyrrolidone (PVP) enhances the polarity of the FePt surface, thereby promoting effective contact between the ZIF‐8 precursors and FePt. The long‐chain molecules of PVP adsorb onto the FePt surface, forming a stable, protective layer that prevents aggregation of FePt particles and creates favorable conditions for the uniform deposition of ZIF‐8. The PEG chains grafted onto the ZIF‐8@FePt surface result in the formation of the final mPNs, which creates a hydrophilic protective layer around the nanoparticles. This layer increases the stability and biocompatibility of the mPNs.

The final synthesized mPNs exhibited a clear core‐shell structure, as confirmed by scanning transmission electron microscopy (STEM) combined with energy dispersive spectroscopy (EDS) mapping of a single mPN, which showed the distinct distribution of Zn, Fe, and Pt (Figure 2B(i)), well following the intended design. Figure 2B(ii,iii) presents the selected area electron diffraction (SAED) patterns of the ZIF‐8 shell and the FePt core, respectively. The well‐defined ring nature of the SAED pattern confirms the formation of crystallites of ZIF‐8 (Figure 2B(ii)). Because of the relatively small crystallite size, the rings and diffraction spots are diffuse and unclear (Figure S1A, Supporting Information). In contrast, the high‐resolution transmission electron microscopy (HRTEM) image of the FePt core demonstrates clear and distinct diffraction spots, confirming the formation of a crystalline structure (Figure 2B(iv)). Based on the STEM and EDS results (Figure S1B–E, Supporting Information), the Fe and Pt elements are present in approximately a 1:1 ratio, with Fe accounting for 53.6% and Pt for 46.4%. The nearly equal distribution of Fe and Pt atoms is characteristic of the L1_0_ phase, which is crucial for locomotion requiring strong magnetic properties.^[^ ^19^ ^]^

We measured the magnetic properties of the synthesized FePt, MMP, and Fe_3_O_4_ NPs, using a vibrating sample magnetometer (VSM). We used Fe_3_O_4_ NPs for comparison because they are the most commonly used biocompatible magnetic nanoparticles for microrobotic platforms.^[^ ^26^ ^]^ The hysteresis loops show that the MMP reaches an intrinsic magnetization maximum of 56.11 emu g^−1^ at the maximum field of 1800 mT (Figure 2C), and the remanent magnetization is ≈24.70 emu g^−1^. In comparison, FePt NPs have an intrinsic magnetization maximum of 54.06 emu g^−1^ and a remanent magnetization of 29.75 emu g^−1^. These results indicate that both MMP and FePt NPs exhibit hard magnetic properties. In contrast, the Fe_3_O_4_ NPs exhibit a soft magnetic response, with both their intrinsic magnetization and remanent magnetization of 0 emu g^−1^ and 0 mT, respectively. Notably, the coercive field of MMP is slightly above 153.68 mT, while FePt NPs exhibit a significantly higher coercivity of 492.13 mT, an order of magnitude greater than typical strong permanent ferromagnetic elements such as cobalt and nickel.^[^ ^20^ ^]^

Based on the magnetic characteristics of MMP, the heat energy generated by magnetic FePt nanoparticles can be controlled by adjusting the applied magnetic field. This is because heat generation primarily originates from power losses associated with Brownian and Néel relaxation mechanisms (Note S3 and Figure S2, Supporting Information). We use external radiofrequency (RF) heating to facilitate the accelerated release of drugs, such as doxorubicin (DOX). This technique uses the increase in local temperature to either modify the structure of the drug carrier or enhance the diffusion of drug molecules from it, thereby enabling rapid drug release. Our approach explicitly focuses on evaluating the temperature response of MMP when exposed to RF. The MMP solution (10 mg mL^−1^) is exposed for up to 10 minutes to an RF field at a frequency of 338 kHz, generated by an RF heater placed at a distance of 5 cm. The RF heating induces a temperature increase of the MMP solution (10 mg mL^−1^) in a tube from room temperature to 46 °C and finally to 57.2 °C over 30 minutes (Figure 2D; Figure S3A, Supporting Information).

X‐ray diffraction (XRD) analysis is also performed on the MMP, FePt, and ZIF‐8 nanoparticles in Figure 2E. The XRD patterns of FePt NPs and MMP show the presence of peaks at (110) and (111), indicating that the core FePt NPs have a high order. This indicates that the synthesized FePt NPs undergo the rearrangement of Fe and Pt atoms into a long‐range chemically ordered face‐centered tetragonal structure without the need for high‐temperature annealing. Thus, the XRD pattern of the MMP shows characteristic peaks associated with ZIF‐8 and FePt, highlighting the composite nature of the MMP and confirming the integration of these components.

Besides having unique magnetic properties, it is also necessary for MMP to possess enhanced drug‐loading capacity. The used ZIF‐8, a material known for its high porosity and surface area, has a pore size of 1.24 nm measured by the Brunauer‐Emmett‐Teller (BET) test (Figure 2F). The N_2_ adsorption‐desorption isotherms show that both the ZIF‐8 and ZIF‐8@FePt exhibit typical I‐type isotherms, which is a characteristic of microporous materials, indicating that they are typically microporous materials (Figure S3B, Supporting Information). The BET surface area of ZIF‐8@FePt is ≈712.68 m^2^ g^−1^ (Figure S3C,D, Supporting Information). X‐ray photoelectron spectroscopy (XPS) is used to characterize the elements present in the sample to show the relationship between the FePt and ZIF‐8. Figure S4 (Supporting Information) shows the complete XPS spectrum of the core‐shell ZIF‐8@FePt. Zn 2p, Pt 4f, Fe 2p, C 1s, O 1s, and N 1s can be observed from the entire XPS spectrum. Also, the exemplary spectra of Zn 2p and Pt 4f prove the existence of the divalent Zn and Pt elements in the synthesized MMP (Figure S4C,F, Supporting Information). As mentioned during the synthesis process of MMP, the PEG coating significantly increases the zeta potential up to −42 mV, indicating that the MMP possesses high stability in Phosphate‐buffered saline (PBS) solution (Figure 2G).

### 2.3 Magnetic Propulsion and Reconfigurable Swarm Behaviors

**Figure 3:**
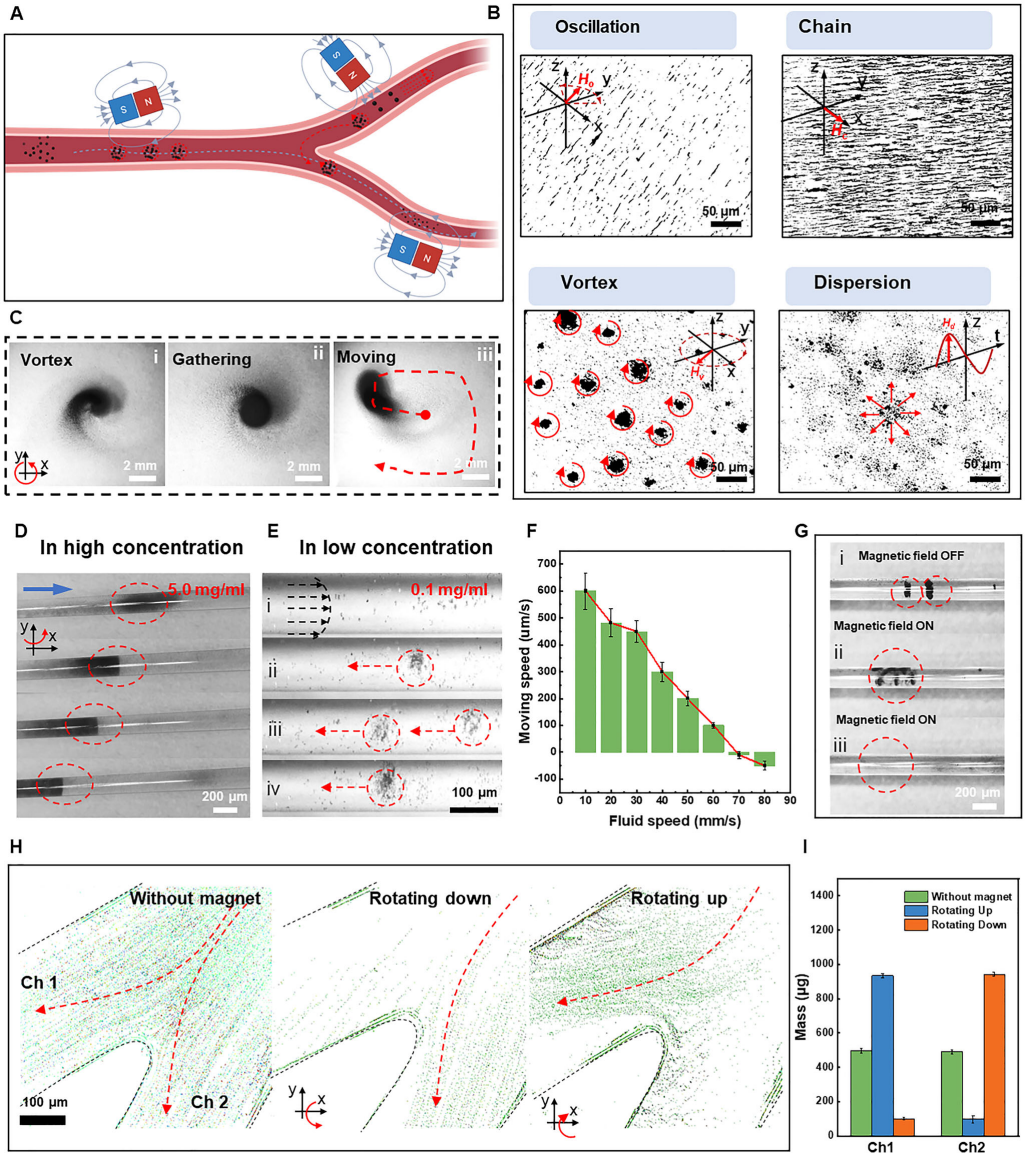
Magnetic propulsion and swarm behaviors of the MMP in a blood vessel phantom. A) A schematic illustration of the swarm behaviors of the MMP in an artificial blood vessel phantom with vessel branches. B) Snapshots from the top view show reconfigurable swarming behaviors of oscillation, chain, vortex, and dispersion in the Helmholtz coil system. C) The vortex, aggregation, and movement behaviors of the MMP swarms under a rotating magnetic field. D,E) The upstream behavior of the MMP at different concentrations. The blue arrow indicates the downstream direction. F) The movement speed of the MMP at different flow velocities. G) The MMP aggregates can quickly disperse under a high‐speed rotating magnetic field (with the magnet rotating axially along the pipeline). H) A rotating magnetic field controls MMP to select different channels at a vessel branch. The colored points stand for the MMP. I) The mass fraction of the MMP passes through under different rotating magnetic fields.

Compared to single magnetic particles, a swarm of mPNs can produce stronger propulsion forces and is capable of easier navigation control, easier tracking, and enhanced drug delivery. We schematize the magnetic field responsiveness, reconfigurable swarm behavior, and the ability of MMP to move upstream of blood vessels in **Figure** **3**A. First, several different patterned magnetic fields are designed (Note S4 and Figure S5A,B, Supporting Information). Then, the response of MMP (1 mg mL^−1^) to the weak magnetic fields in a PBS solution is validated. The MMP rapidly aligns with the patterned magnetic field within a few seconds, as shown in (Figure S5B and Movie S1, Supporting Information).

To more precisely and dynamically control the collective behavior of MMP (see the locomotion mechanism in Figure S6 and Note S4, Supporting Information), we designed a Helmholtz coil system (Figure S6D, Supporting Information). The externally applied rotating magnetic field, circularly polarized in any 3D plane, enables reconfigurable swarm behaviors. Four different swarm behaviors of MMP, namely oscillation, chain, vortex, and dispersion, are observed under the magnetic field generated by the Helmholtz coils (Figure 3B). When subjected to a magnetic field ***H_o_*** *(t) = H_o_* [*cos(ωt)* ***e*** *_x_ − sin(ωt)* ***e*** *_y_*] oscillating at an angle of 90 degrees in the x‐y plane, the MMP rapidly oscillates in the form of chains. In a magnetic field along the x‐direction, ***H_c_*** *(t)) = H_c_cos(ωt)* ***e*** *_x_*, the magnetic torque ***T_m_*** *= µ_0_ (* ***m*** *×* ***H_c_***) induces the particles to align into chains that follow the direction of the magnetic field, where *µ_0_* is the vacuum permeability (4π × 10^−7^ H m^−1^), and ***m*** indicates the magnetization of the MMP (Figure 3B(i)). Under the external rotating magnetic field ***H_v_*** *(t) = H_v_* [*cos(ωt)* ***e*** *_x_ − sin(ωt)* ***e*** *_y_*], the spinning MMP generates a small vortex fluid field with itself as the vortex core (Figure 3B(ii)). The spinning‐induced hydrodynamic interaction and the time‐averaged magnetic dipole forces attract the particles to approach each other, and short‐range repulsion keeps them from getting too close to one another. Thus, a vortex is formed as more particles continue to aggregate (Figure 3B(iii)). When the oscillating magnetic field ***H_d_*** *(t) = H_d_* [*sin(*ω*t)* ***e*** *_z_*] is applied, the particles suddenly disperse from their original patterns and gradually form a uniform distribution (Figure 3B(iv); Movie S4, Supporting Information). The vortex‐to‐dispersion transformation exhibits a distinct fireworks‐like behavior that lasts several seconds until all particles are dispersed.

Considering the magnetic strength limitations and spatial constraints of Helmholtz coils, the collective motion of the MMP can also be controlled by a rotating permanent magnet (NdFeB, cube, 10 × 10 × 10 mm^3^), which actuates the MMP in a 2D plane (Figure S5(C,D) and Movie S2, Supporting Information). Observing the actuation patterns of the particle swarm under an optical microscope as it rolls forward under a 10 mT rotating magnetic field, we measured the motion trajectories and instantaneous velocities of the MMP, obtaining a maximum velocity of 632 ± 45 µm s^−1^ (Figure S5D,E, Supporting Information). In addition to the linear movement, MMP shows a collective vortex behavior under the rotating magnetic field parallel to the 2D plane (Figure 3C(i); Figure S5(F) and Movie S3, Supporting Information). Furthermore, we can generate magnetic trapping at a specific location to improve the visibility of the MMP under MSOT imaging. As the aggregation time increases, the aggregated particles form a disc shape and can move with the applied magnetic field (see Figure 3C(ii,iii)).

In order to adapt to the complexities of the cerebral vasculature and to enable MMP to deliver drugs to their target sites, it is essential to perform tests that simulate the behavior of MMP swarms in blood flow, including upstream migration, dispersion, and selection of target vascular channels. The presence of a wall allows a particle to break the flow rate and pressure symmetry around itself in a viscous fluid, which helps convert rotational movement into translational movement, thereby inducing pure propulsion. MMP swarms tend to aggregate and increase in size to gain greater propulsive forces as they move upstream to cope with the shear force and drastic impact (Figure 3D; Figure S5G, Supporting Information). The swarm is confirmed as a continuously expanding disk, rolling upstream against the wall from the top view (Figure 3E; Movie S5, Supporting Information). These MMP swarms can move against the flow with an average velocity of 20 µm s^−1^ under a fluid velocity of 70 mm s^−1^ under the rotating magnetic fields (Figure 3F; Figures S7–S9, Supporting Information). As illustrated in Figures S6,S8, and S9 (Supporting Information), we employed multiple magnetic setups, including a permanent magnet, a Halbach array, and programmable electromagnetic coils, to provide different field strengths, gradients, and spatial configurations for actuation and control.

However, MMP aggregates easily due to their inherent magnetism, and it is necessary to disperse them after they reach their target site to avoid the risk of vascular occlusion. An axially rotating magnetic field is applied that rapidly rotates along the direction of the tube (>1000 rpm) to disperse the aggregated MMP under centrifugal force (Figure 3G; Movie S6, Supporting Information). When MMP is actuated in the Y‐shaped junction, the MMP can selectively choose their respective paths under the driving force of the rotating magnetic field (Movie S7, Supporting Information). In the absence of a magnetic field, MMP flows uniformly and randomly toward both channels under the influence of fluid flow (Figure 3H(i)). However, when the rotating magnetic field is directed toward channel 1 (CH1), MMP predominantly flows toward CH1 (Figure 3H(ii)); similarly, when the magnetic field is diverted toward channel 2 (CH2), MMP predominantly flows toward CH2 (Figure 3H(iii)). Therefore, by changing the external magnetic field, we can control the upstream mPNs, disperse them, and select appropriate channels to transport drugs to the targeted sites. The efficiency of drug transport to such sites is calculated to be 90.5 ± 3.26% under magnetic field control by collecting MMP from CH1 and CH2 (Figure 3I).

### 2.4 In Vitro Evaluation of the MMP

**Figure 4:**
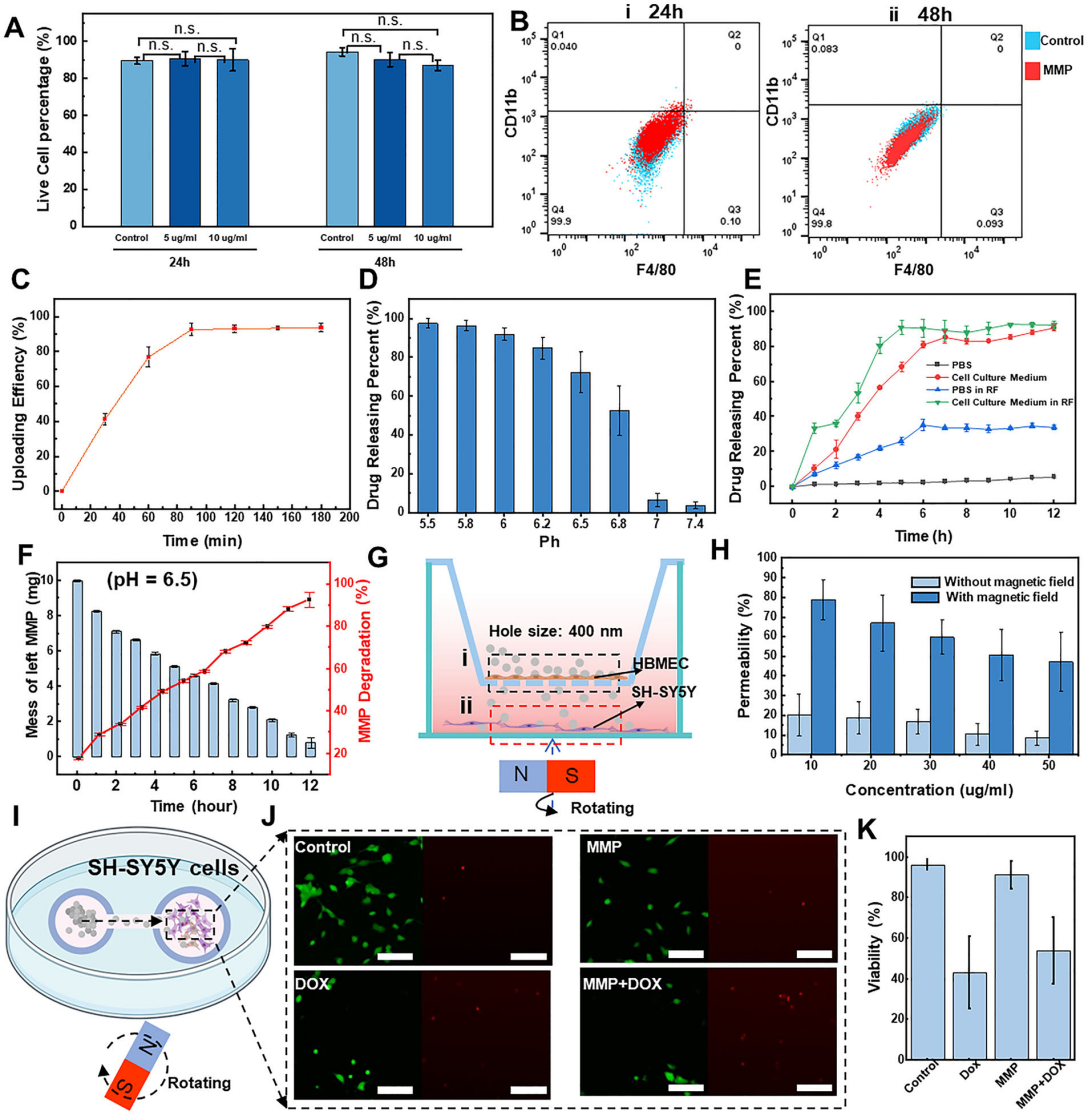
In vitro evaluation of the MMP. A) The biocompatibility of different concentrations of the MMP for 24 and 48 h using the SH‐SY5Y cells. B) The flow cytometry results of F4/80 and CD11b, which are inflammatory markers for macrophages, expression on control and the MMP at 24 and 48 h using J774A cells. C) The loading efficiency of doxorubicin (DOX) under different loading times. D) DOX release rate of the MMP in PBS solutions with varying pH levels. E) The effect of RF heating on DOX drug release in PBS and cell culture medium. F) Degradation of the MMP in a cell culture medium with a pH value similar to that of the tumor microenvironment (pH = 6.5). G) Under the rotating magnetic field, the aggregation and movement states of the MMP in the regions of (i) and (ii). H) Comparison of the penetration capacity of the MMP across an artificial blood–brain barrier with and without a rotating magnetic field. I) Schematic demonstration of the MMP crossing the channel to the SH‐SY5Y cells area to release the DOX drugs. J) Calcein‐AM (Green) and Ethidium Homodimer‐1 (Red) microscopy images of the SH‐SY5Y cells after DOX‐loaded MMP. Red indicates dead cells and green indicates live cells. The scale bar is 100 µm. K) The effect of the MMP carrying and releasing DOX on SH‐SY5Y cells after 48 hours.

The biocompatibility of MMP was tested using SH‐SY5Y neuroblastoma cells in Dulbecco's modified Eagle's medium (DMEM) containing various concentrations of MMP. After 48 hours at a concentration of 10 µg mL^−1^, the cell viability remained as high as 87 ± 2.77% (**Figure** **4**A; Figure S10, Supporting Information). We also used macrophages to investigate the immune response against MMP and evaluated its immunocompatibility. Macrophages can adopt two major phenotypes in response to environmental stimuli: the classically activated pro‐inflammatory (M1) and the alternatively activated anti‐inflammatory (M2) macrophages, which can be observed by flow cytometry using surface markers, such as CD11b and F4/80. Initially, there is a slight increase in the expression of factors associated with the M1 phenotype upon introducing MMP, indicative of a foreign body rejection response (Figure 4B(i); Figure S11, Supporting Information). After 48 hours, the response decreases to the native surface expression levels, signaling that the MMP does not induce an inflammatory response in macrophages (Figure 4B(ii); Figure S11, Supporting Information). This non‐inflammatory behavior of macrophages implies that MMP has sufficient compatibility with the immune system, thanks to the non‐inflammatory nature of the PEG/ZIF‐8 combination, allowing its stable presence in the bloodstream.

The drug carrier potential of MMP is investigated with a chemotherapeutic drug agent, DOX, effective against several types of cancer, to explore its applicability in tumor environments. The fluorescent properties of DOX allow the quantification of its concentration by measuring the fluorescence intensity of the solution (Figure S12, Supporting Information). DOX loading into MMP was monitored over time and reached a plateau at ≈9 hours with an encapsulation efficiency of 93.9 ± 2.65% (Figure 4C).

The release of DOX is facilitated under slightly acidic conditions by adjusting the pH to the intratumoral environment. As shown in Figure 4D, the drug release rate reached 97.43% at a solution pH of 5.5. This enhanced drug delivery by MMP at low pH values is particularly effective in acidic environments, such as those found in tumors or infection sites. To investigate the effect of RF heating on drug release, we placed MMP in PBS (pH = 7.4) and cell culture medium (pH = 6.8). Results indicate that applying RF significantly accelerates drug release, especially in PBS solution (Figure 4E). That's because the magnetic FePt nanoparticles within MMP generate heat under RF exposure (Note S3, Supporting Information), promoting the diffusion and release of drug molecules.^[^ ^25^ ^]^ In addition to drug release kinetics, degradability is a critical issue for the biocompatibility of nanoparticles. The MMP was placed in a cell culture medium with a pH value similar to the tumor microenvironment (pH = 6.5) to study its degradability. The nanoparticles are collected by centrifugation, dried, and weighed at different times to calculate the degradation rate, which indicates that the degradation rate of MMP reaches 92.7 ± 3.54% after 12 hours (Figure 4F). Taken together, the results of our study and previous studies suggest that MMPs exert a greater degree of control over drug delivery properties due to the synergistic increase in drug release rate resulting from the combined application of pH changes and RF heating mechanisms.^[^ ^27^ ^]^

The MMP has the potential to cross the blood–brain barrier (BBB) to deliver drugs to brain tumor sites. In this study, a transwell migration assay through a confluent layer of human brain microvascular endothelial cells (HBMEC) seeded with a 0.4 µm pore size membrane is conducted to simulate the blood–brain barrier (BBB) model,^[^ ^28^ ^]^ and the ability of MMP passage through the BBB model is evaluated (Figure 4G). In addition to the HBMECs on the transmembrane, SH‐SY5Y neuroblastoma cells are seeded on the bottom chamber to mimic the brain tumor microenvironment with the BBB. The MMP crossing is facilitated by using a rotating magnetic field to accelerate the passage of MMP through the BBB model. First, the MMP is distributed on the upper surface of the BBB membrane with HBMECs, and then they pass through the BBB transwell membrane to the lower chamber with SH‐SY5Y neuroblastoma cells within two hours when we apply a rotating magnetic field (Figure S13, Supporting Information). MMP effectively moves across the transwell after 6 hours of rotating magnetic stimulation, and the transport ratio of MMP at 10 µg mL^−1^ reached 78.9 ± 10.25% (Figure 4H), whereas the transport ratio without the magnetic field was only 20.14 ± 10.64%.

After crossing the BBB, the next and final step of drug release is the therapeutic efficiency for the tumor model. We designed a dual‐chamber setup on a cell culture dish, with one chamber for the introduction of MMP and another for the culture of SH‐SY5Y neuroblastoma cells. A rotating magnetic field facilitates the transport of MMP into the chamber containing SH‐SY5Y cells (Figure 4I). During this cell culture experiment, the acidic environment of the MMP induces the degradation of the ZIF‐8 shell, triggering the release of the encapsulated drug. MMP successfully releases the DOX and achieves cell mortality rates of 53.9 ± 16.3% within 24 hours, comparable to the 43.2 ± 17.9% observed with direct DOX administration (Figure 4J,K). In contrast, MMP without the drug exhibited significantly lower cell death rates compared to the control group, underscoring the ability of MMP to effectively transport and release drugs in the acidic tumor microenvironment, thereby fulfilling the ultimate goal of targeted drug delivery (Figure 4J).

### 2.5 Real‐Time MSOT Imaging of MMP Ex Vivo

**Figure 5:**
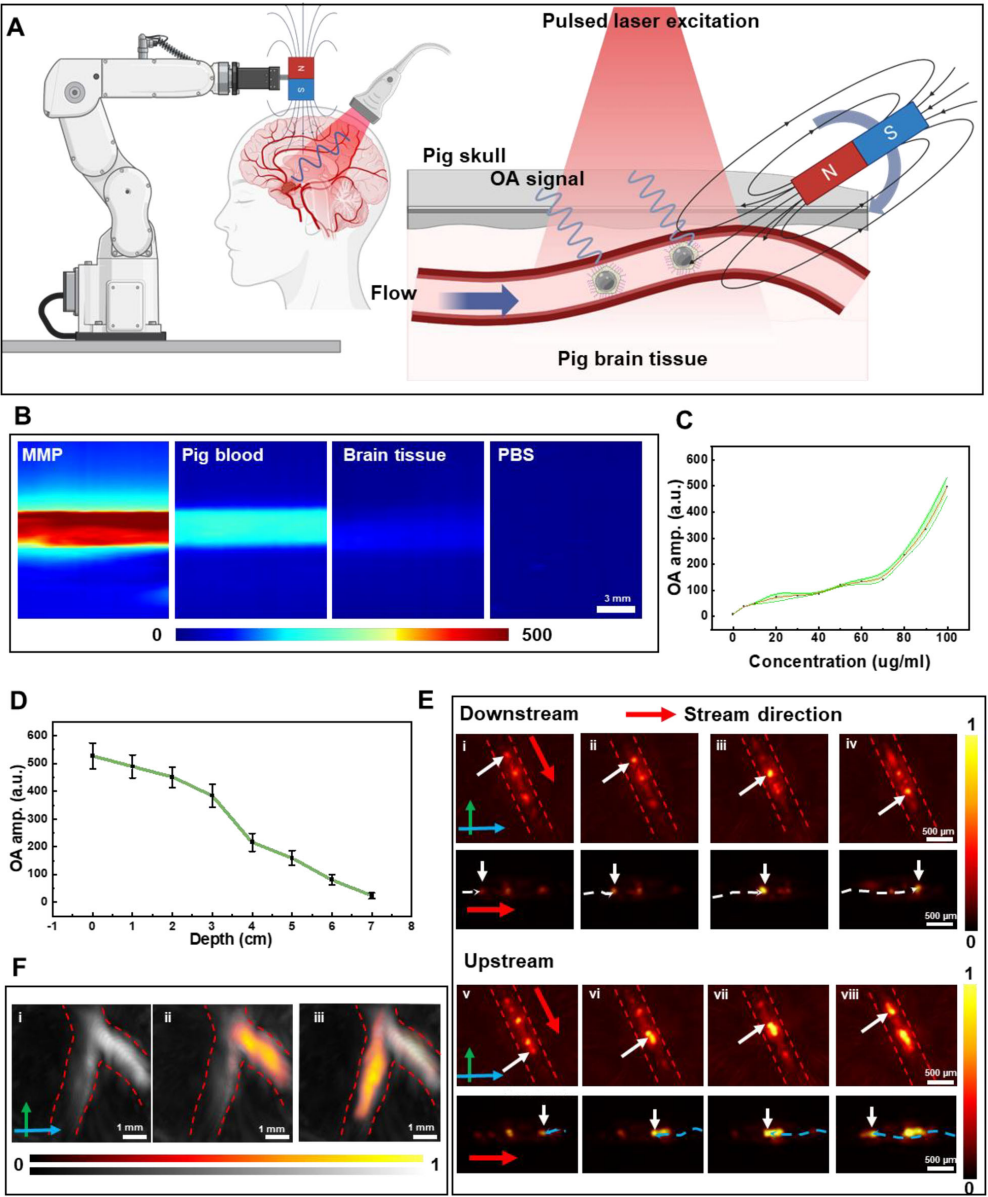
Real‐time tracking and magnetic manipulation of the MMP inside ex vivo porcine models. A) Schematic illustration of using the robotic arm holding a permanent magnet to control the MMP swarm behaviors, and schematic of the imaging procedure used to track the MMP in the tube under the thin porcine cranial bone. B) MSOT images of the MMP, porcine blood, brain tissue, and PBS in plastic tubes with laser wavelengths at 920 nm. C) The corresponding MSOT signal amplitude of the MMP with different concentrations. D) Dependence of the MSOT signal amplitude of the MMP on its depth in tissue. E) Under the control of a rotating magnetic field, the MMP aggregates and moves upstream in a flowing channel. The green arrow represents the y‐axis, the blue arrow represents the x‐axis, and the yellow arrow represents the z‐axis. F) The rotating magnetic field guides the MMP in selecting different pathways at vascular bifurcations. The green arrow represents the y‐axis, and the blue arrow represents the x‐axis.

Effective clinical use of MMP requires precise navigation and real‐time imaging within deep tissues (Note S5, Supporting Information). Porcine brain tissue is used to simulate the human brain, 100 µm‐diameter polydimethylsiloxane (PDMS) soft tubes are used as brain blood vessels, and MSOT is used for real‐time imaging and tracking of MMP (**Figure** **5**A). MMP has higher optical absorption than the blood background, so that it can be tracked in real‐time for deep tissue imaging in vivo. We measure the signal amplitudes of the MMP versus pig blood to evaluate their suitability for MSOT imaging (Figure S14A,B, Supporting Information). The MMP exhibits strong MSOT contrast at the NIR wavelengths, particularly at 920 nm, where blood absorption in mammalian tissues is reduced, allowing for the deepest tissue penetration. The MSOT contrast of MMP is examined in various background materials, including PBS, porcine blood, brain, and bone at 920 nm, and shows that MMP exhibits strong contrast on top of these backgrounds (Figure 5B). The contrast further increases with the concentration of the MMP (Figure 5C; Figure S14C, Supporting Information).

Considering the real‐world application scenario of tracking MMP through a thick tissue, a plastic tube filled with MMP PBS solution is placed under the soft brain tissue at different depths ranging from 0 to 7 cm. MSOT can image the MMP embedded as deep as ≈7 cm inside the tissue (Figure 5D), demonstrating its high resolution and contrast for deep tissue monitoring of the microrobots. While ultrasound imaging cannot image the MMP in the center of the bone, MMP is detectable with MSOT (Figure S14D,E, Supporting Information). These experiments demonstrate that MMP can be imaged through soft and hard tissues by MSOT.

Under physiological conditions, blood is constantly flowing; hence, MMP must adapt to the flowing blood environment and may even require the ability to move against the blood flow. Under the influence of a magnetic field, MMP aggregates to enhance its MSOT signature and follow the flow (Figure 5E(i–iv)). Then, by changing the direction of the magnetic field to rotate against the direction of fluid flow, the aggregated MMP can move upstream against the blood flow (Figure 5E(v–viii)); Movie S8, Supporting Information). In the Y‐structure vessel, as a typical structure in the complex cerebrovascular system, the MMP is controlled to choose the right channel for targeted drug delivery (Figure 5F). Similarly, we can create magnetic trapping at a specific location to enhance the visibility of the MMP in MSOT imaging, and the MMP is injected into the superficial capillaries of the porcine cortex and is tested for clearance under syringe pressure (Figure S14F, Supporting Information). The rotating magnetic field induces the MMP to aggregate and causes them to move upward in venous vessels (Figure S14G and Movie S9, Supporting Information). These experiments demonstrate that MMP can be observed in their collective behavior in deep tissues, ensuring that drugs can be delivered to specific sites.

We further explored the dual‐modality imaging capabilities of the MMP to show detectable contrast not only in MSOT but also in MR imaging. After injecting MMP into the anterior cerebral artery of the pig brain, they become visible on MR and can be precisely localized within the brain tissue (Figure S15A, Supporting Information). The T2‐weighted MR images at different MMP concentrations, with color‐coded T2 maps illustrating a pronounced contrast effect, show the concentration‐dependent signal enhancement (Figure S15B, Supporting Information). Finally, quantification of the relationship by plotting the transverse relaxivity rate (R_2_ = 1/T2) versus MMP concentration reveals a linear correlation, highlighting the potential of MMP as a highly effective MR contrast agent (Figure S15C and Table S2, Supporting Information). These findings underscore the utility of MMP as a versatile multimodal imaging agent and provide a robust basis for precise imaging applications.

### 2.6 In Vivo MSOT Imaging of the Circulating MMP in Murine Vasculatures

**Figure 6:**
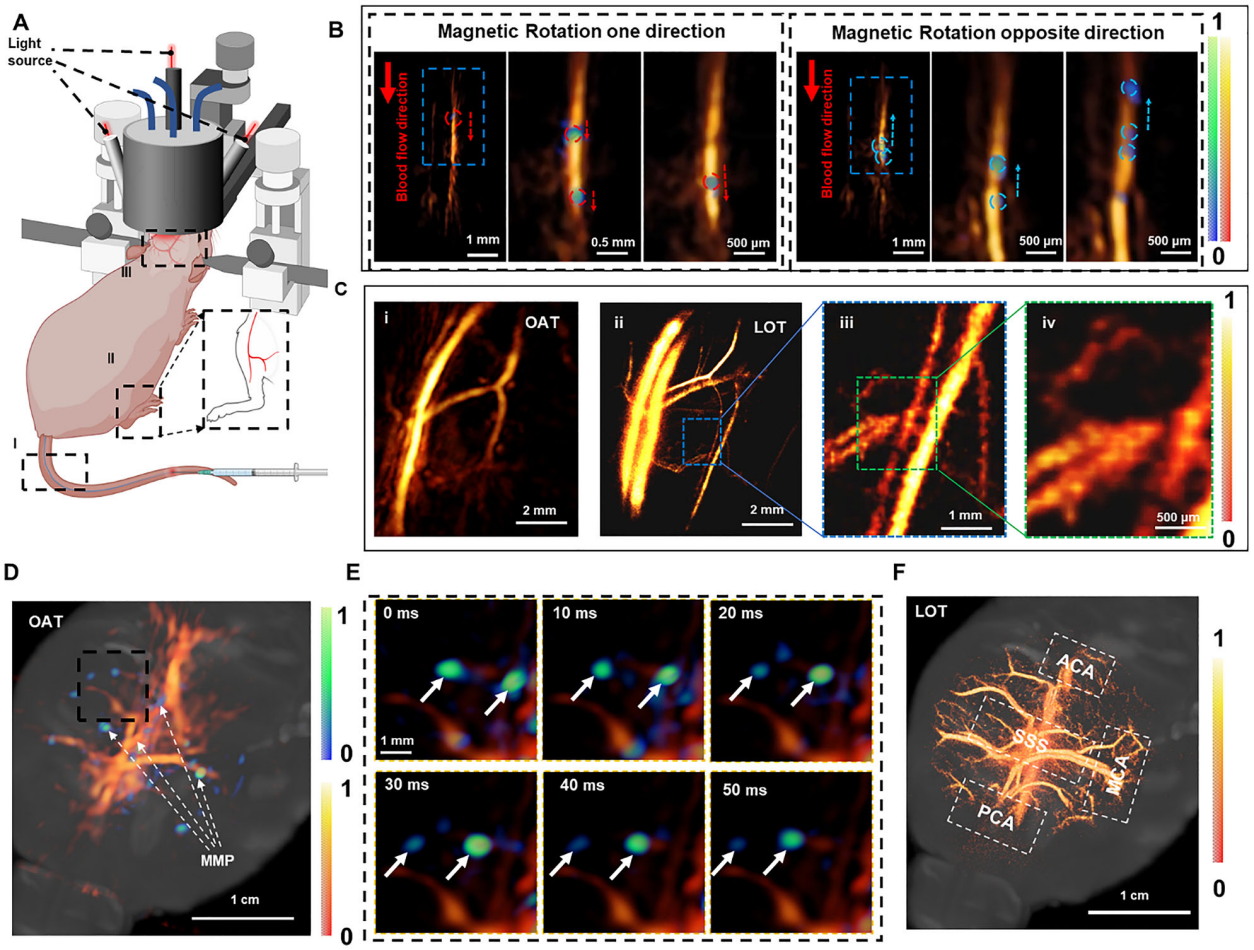
Noninvasive MSOT imaging of the MMP circulating in the mouse tail, leg, and brain in vivo. A) Schematic description of the microrobot injection procedure into the tail and OAT imaging at the tail, leg, and brain in vivo. The MMP is transported with the natural blood circulation from the tail into the mouse legs and brain, continuously monitored inside the field of view (FOV) using volumetric MSOT. B) The MMP in the tail can actuate upstream against the blood flow by the rotating magnetic field. And MSOT images of the MMP in the right leg vessels. C) Localization of the MMP in the mouse leg vessels using the localization optoacoustic tomography (LOT) technique. D) MSOT images of the mouse brain superimposed on the differential (background‐subtracted) images. E) A magnet was then used to manipulate the MMP inside the blood‐filled mouse brain. MSOT images of the MMP in the brain. F) Maximum intensity projections of the 3D images rendered with LOT. Abbreviations: ACA – anterior cerebral artery, MCA – middle cerebral artery, PCA – posterior cerebral artery, SSS – superior sagittal sinus.

In the final step of the study, the suitability of MSOT‐based intravascular 3D real‐time tracking of MMP was investigated in an intact mouse brain vasculature in vivo. As shown in the schematic of the experimental setup (**Figure** **6**A), tail vein injections of MMP in 1 mL PBS solution at a concentration of 1 mg mL^−1^ were performed under MSOT imaging. After injection via the tail vein, the MSOT imaging probe was moved to the tail, where MMP was detected flowing in the bloodstream, and the signal was visible against the blood background. When the rotating magnetic field is activated, the MMP can move against the blood flow, demonstrating the true significance of MMP as it can be controlled and tracked in complex vascular structures (Figure 6B). As shown by the tracking procedure visualized in Movie S10 (Supporting Information), the MMP was injected intravenously through the tail vein and actuated around the caudal region. After arterial injection, MSOT was used to acquire images of the mouse's right leg region. Enhanced spatial resolution can further be achieved by analyzing ≈6000 consecutive image volumes using localization optoacoustic tomography, as shown in the dorsal view of the mouse leg vasculature (Figure 6C(i,ii)). Thus, the MMP significantly improves the quality of vascular imaging, allowing more precise visualization of the blood vessels (Figure 6C(iii,iv)). This experiment demonstrates the potential of MMP to improve the contrast, spatial resolution, and penetration depth of MSOT imaging.

The feasibility of in vivo trapping and controlling the motion of MMP in the bloodstream under MSOT guidance is then demonstrated in the mouse brain. Volumetric 3D imaging of the mouse brain is achieved with the spherical array MSOT probe pointing downward and the enclosed volume filled with deionized water (Figure 6A). In this way, major cerebral vessels can be clearly visualized during the intravenous injection of the MMP. Singular value decomposition (SVD) filtering is applied to a sequence of MSOT images to separate the slowly varying vascular background signal from the rapidly moving spheroids associated with MMP flowing in the bloodstream (Figure 6D,E). The signal from MMP diminishes as they move into deeper brain areas, as can be visualized in Movie S11 (Supporting Information). Vessel visibility and spatial resolution of the images are significantly improved with the LOT technique, which clearly identifies the ACA, MCA, the posterior cerebral artery (PCA), the superior sagittal sinus (SSS), and other pial vessels (Figure 6F; Figure S16 and Movie S11, Supporting Information). Specifically, vascular structures separated by 22 µm can be resolved by LOT over a depth range of ≈1.5 mm from the brain cortex, which is ≈2.5 mm away from the skin surface, through the intact ≈0.5 mm‐thick scalp and 0.4–0.6 mm‐thick skull.^[^ ^29^ ^]^ Taken together, our results indicate that the force induced by a permanent magnet is sufficient to move the MMP in the bloodstream of the mouse. The MMP is further shown to enhance the ability of MSOT to visualize finer cerebral vasculature while providing a powerful in vivo monitoring capability for microrobots.

## 3 Discussion

In this study, we built and demonstrated the advantages of the multifunctional magnetic microrobot platform for targeted drug delivery, which addresses the most frequent issues for the nanocarrier‐based drug delivery systems, including drug loading capacity, anatomical targeting specificity, controlled and sustained release, and real‐time optoacoustic tracking of the carriers.^[^ ^30^ ^]^ This microrobotic platform can be fabricated using bottom‐up methods in high throughput, can carry a high volume of medications, thanks to its ZIF‐8 shell, and can move in complex vasculature models, thanks to its magnetically programmable and biocompatible hard magnetic FePt core. Compared to previous FePt‐based microrobots, MMP has a smaller dimension (≈200 nm), which enables it to pass through capillaries.^[^ ^20^ ^]^ The MMP, which is also investigated in vivo in this study, is the first realistically designed magnetic microrobotic platform with a combination of hard magnetic metallic core (FePt) and metal–organic frameworks‐based shell. MMP can serve as a unique drug delivery platform with a high drug‐loading capacity and further exhibit strong contrast under real‐time 3D MSOT guidance, which can greatly facilitate efficient drug transport to the target sites through reconfigurable swarms and controllable drug release, addressing a long‐standing challenge. We synergistically designed and engineered FePt@ZIF‐8 mPNs to assemble the MMP, thereby overcoming the inherent limitations of each material and endowing the platform with magnetic propulsion and high drug‐loading capabilities. We then demonstrated the reconfigurable swarm behavior of MMP in various forms, such as oscillation, chain, vortex, and dispersion, to adapt to different vascular environments, thereby enhancing drug transport efficiency. The swarm behavior of MMP also enhances their propulsion, enabling them to navigate against the blood flow. Furthermore, our ex vivo and in vivo experiments demonstrate that the MMP shows strong MSOT contrast, thus enabling real‐time imaging feedback and significantly improving vascular imaging quality.

The MMP design, with engineered nanoparticles of FePt core and ZIF‐8 shell, demonstrates precise remote control capabilities, efficient drug loading, and precise release capabilities. The ZIF‐8 coating contributes to increased drug loading capacity. However, high drug loading is not a prerequisite for clinical benefit, but likely better selective targeting and improved on‐ versus off‐target drug accumulation, among others. Compared to previously reported drug delivery systems, the MMP not only achieves higher drug loading capacity but also realizes more accurate navigation in complex vascular environments through reconfigurable swarm behavior by integrating biocompatible and hard magnetic FePt core.^[^ ^23^, ^31^ ^]^ Although the magnetic control of the MMP has been investigated in detail using both Helmholtz coils and permanent magnets, it is difficult to conclude that magnetic torque or magnetic gradient is solely responsible for the magnetic actuation of the MMP.^[^ ^32^ ^]^ The detailed magnetic characterization of the swarm behavior of the MMP requires further investigation due to the compound effect of magnetic fields being hard to analyze on the microscale.

In addition, the complete degradation of the ZIF‐8 shell and FePt core in the mildly acidic tumor environment for drug release, a feature of this design, reduces the risk of long‐term residues in the body, thereby enhancing the safety of the treatment.^[^ ^33^, ^34^ ^]^ In this way, although each microrobot is ≈200 nm in size and therefore incompatible with renal clearance, the remaining FePt cores, which are less than 10 nm in size, can be eliminated from the body through the degradation of the ZIF‐8 shell, without causing any further complications.^[^ ^35^, ^36^ ^]^ Furthermore, MSOT enabled noninvasive imaging and tracking of MMPs deep within living soft tissues, an aspect not explored in previous studies, marking a significant advance in real‐time monitoring capabilities. However, due to the resolution limits of current biomedical imaging modalities and the challenges in quantifying drug concentrations within the complex vasculature of the rodent brain,^[^ ^37^ ^]^ in vivo demonstration of drug delivery with MMP was beyond the scope of this study.

Despite the progress made in this study, future work will focus on potential clinical translation aspects of the MMP. While their biocompatibility has been studied in vitro, their ability to cross the blood–brain barrier has yet to be thoroughly evaluated in vivo. Although in vivo experiments for brain imaging and tracking of MMPs have been conducted in a limited number of animal models, the processes of drug release and the therapeutic effects on tumors require further investigation. However, the spatial and temporal resolution of current biomedical imaging modalities remains insufficient to simultaneously resolve the individual trajectories and collective swarm dynamics of magnetic microrobots at low concentrations in deep biological tissues. As emphasized by Langer and colleagues, ensuring the long‐term biocompatibility of nanomaterials is crucial for their translation into clinical applications.^[^ ^30^ ^]^ Therefore, the long‐term biocompatibility, stability, and functionality of MMP, including circulation time and organ distribution in complex biological environments, should be thoroughly evaluated and optimized under in vivo conditions. To facilitate the transition of MMP from the laboratory to the clinic, it is imperative to overcome significant challenges associated with translating these technologies from animal models to humans. Although the transwell model used in our study is widely employed to assess blood–brain barrier (BBB) permeability, it cannot fully replicate the dynamic microenvironment, cellular heterogeneity, and complex transport mechanisms of the in vivo BBB models. Advances in medical nano‐/microrobotics require the development of materials with higher biocompatibility and degradability, as well as the enhancement of the intelligence of these nanoparticles, such as autonomous navigation of nanocarriers within the body.

To translate MSOT imaging to the human scale, integration with clinically compatible light delivery systems, such as endoscopic or catheter‐based optical fibers, and adaptation to deeper‐penetrating wavelengths will be essential to overcome tissue attenuation.^[^ ^38^ ^]^ Coupling these advances with optimized acoustic detection geometries could enable high‐resolution, real‐time vascular imaging in large animal models and, ultimately, in human patients. Clinical implementations may be based on endoscopic OA systems or optimized particles incorporating, e.g., a large amount of FDA‐approved ICG to achieve higher sensitivity and thus deeper penetration.^[^ ^39^ ^]^ This study opens a new avenue for the potential clinical use of nano‐/microrobots by exploiting novel materials such as FePt and ZIF‐8. In the subsequent steps, these nanocarriers will be integrated with noninvasive and intraoperative imaging systems and artificial intelligence algorithms to facilitate self‐learning navigation within intricate vascular systems, enabling swarm behaviors and closed‐loop feedback control in the body.^[^ ^40^ ^]^ Through these efforts, we aim not only to advance microrobotic technologies for diverse biomedical purposes but also to open new horizons in shaping the future of precision medicine.

## 4 Experimental Section

### Materials

Hexadecyltrimethylammonium chloride (52366‐10G), hexane (139368‐500ML), oleic acid (O1008‐1G), Iron (III) acetylacetonate (8039120250), platinum (II) acetylacetonate (282782‐5G), oleylamine (O7805‐100G), polyvinylpyrrolidone (PVP, PVP10‐100G), poly(ethylene glycol) (PEG, 202398‐5G), agar (1016151000), methanol (34860‐1L‐R), intralipid (I141‐100ML), and doxorubicin hydrochloride (44583‐1MG) were purchased from Sigma‐Aldrich. Glass coverslips and phosphate‐buffered saline (PBS, pH 7.4) were obtained from Thermo Fisher Scientific. Eudragit L 100–55 acrylic polymer was supplied by Evonik Industries. Silicone tubing with an inner diameter of 0.1 mm was sourced from Dow Silicones. High‐performance instant glue (402, LOCTITE) was purchased from Reichelt Elektronik GmbH. Poly(dimethylsiloxane) (PDMS) elastomer (Sylgard 184, Dow Inc.) was purchased from Biesterfeld Spezialchemie GmbH. Neodymium‐iron‐boron (NdFeB, 5 µm) was purchased from Magquench GmbH.

Human SH SY5Y neuroblastoma cells (ATCC, Cat # CRL 2266; RRID: CVCL_0019), immortalized human brain microvascular endothelial cells (HBMEC; ScienCell; RRID: CVCL_YJ35), and J774A.1 mouse macrophages (ATCC, TIB 67; RRID: CVCL_0358) were purchased for in vitro studies. For flow cytometry, anti‐F4/80 monoclonal antibody (clone BM8; Thermo Fisher Scientific, Cat # 14 4801 82; RRID: AB_10376289) and anti‐CD11b monoclonal antibody (clone M1/70; Thermo Fisher Scientific, Cat # 14 0112 82; RRID: AB_2539241) were used.

### Preparation of Magnetically Programmed Nanoparticles

*Synthesis of FePt Nanoparticles*: FePt nanoparticles were synthesized following a protocol reported by Yildiz et al. with minor modifications.^[^ ^19^ ^]^ FePt nanoparticles were synthesized by dissolving platinum(II) acetylacetonate (0.2 mmol), iron(III) acetylacetonate (0.2 mmol), hexadecyltrimethylammonium chloride (0.8 mmol), and oleic acid (0.5 mL) in 10 mL of oleylamine. The precursor solution was stirred under argon for 1 hour at ambient temperature before being gradually heated to ≈350 °C at a rate of ≈5 °C min^−1^. The temperature was maintained for 3 hours to promote nanoparticle formation. After the reaction mixture had cooled, ethanol was added to precipitate the particles, which were then isolated by centrifugation (8000 rpm, 3 min). The collected FePt nanoparticles were purified through repeated redispersion in hexane and ethanol washing. This purification cycle was performed at least five times to eliminate unreacted precursors and surplus surfactants. After the final centrifugation at 8000 rpm for 3 minutes, the FePt nanoparticles were redispersed in methanol. This step was repeated three times to ensure complete removal of hexane, and the nanoparticles were finally dispersed in 10 mL of methanol for storage and subsequent use. To determine the concentration of FePt, after adequate ultrasonication, 1 mL of the nanoparticle suspension was deposited on a pre‐weighed dry glass slide. Following drying, the mass of the glass slide was measured to ascertain the mass of the FePt nanoparticles, thus enabling the calculation of FePt concentration for future quantitative analyses. Finally, the high‐performance instant glue was dropped onto the dried surface of FePt to secure the FePt nanoparticles. The fixed FePt nanoparticles were then measured for magnetization profile using a vibrating‐sample magnetometer (VSM; model EZ7, Microsense), which was preprogrammed to generate a uniform magnetic field of 1.8 Tesla.

*PVP Modification on the Surface of FePt Nanoparticles*: 10 mg of PVP (average mol wt. 10 000) was weighed and added to 100 mL of methanol solution, yielding a 0.1 mg mL^−1^ concentration. A portion containing 100 mg of FePt from the above solution was added to 50 mL of PVP methanol solution. The mixture was mechanically stirred (using a Polytetrafluoroethylene (PTFE) stirrer at a speed of 800 rpm and a temperature of 50 degrees Celsius) for 24 hours. After centrifugation at 8000 rpm for 3 minutes, the supernatant was decanted, and the precipitate was subjected to ultrasonication in 10 mL of methanol for 5 minutes to ensure uniform dispersion. This process was repeated at least five times to remove free PVP molecules. Finally, the dispersion was transferred into 10 mL of methanol solution and sealed for storage in a refrigerator at 4 °C.

*In situ Synthesis of ZIF‐8 on the Surface of FePt Nanoparticles*: In a 10 mL methanol solution, 202 mg of zinc nitrate hexahydrate was dissolved with stirring for 30 minutes until fully dissolved, referred to as Solution “A”. In a separate beaker with 10 mL of methanol, 130 mg of 2‐methylimidazole was added and stirred until fully dissolved, labeled as Solution “B”. Then, 10 mL of a PVP‐modified FePt methanol solution was taken. At this stage, 1 mg of the Doxorubicin hydrochloride drug was added if subsequent in vivo optoacoustic imaging experiments or in vitro drug release studies were planned. It's important to note that if the drug is added at this point, all following steps should be performed away from light, and the synthesized MMP must be used within 24 hours since the DOX molecule is unstable at higher temperatures and at acid or alkaline pH, and should be protected from light. Next, 10 mL of Solution “B” was added, thoroughly mixed, and sonicated for 10 minutes to ensure even drug dispersion. Solution “B,” which contained the drug and FePt, was slowly introduced into Solution “A” through a dropping funnel over 5 minutes. The reaction mixture was then stirred continuously at room temperature for 24 hours.

Subsequently, the mixture was centrifuged to remove the supernatant partially, and 10 mL of methanol was added, followed by sonication. The solution was placed on a strong magnet for 10 minutes, and half of the supernatant was removed. This step was repeated five times to eliminate unreacted substances (zinc nitrate hexahydrate and 2‐methylimidazole) and any ZIF‐8 that did not synthesize on the FePt surface. Following a second centrifugation step, the supernatant was carefully decanted, and the resulting precipitate was resuspended in 10 mL of PBS buffer. This procedure was repeated five times, ultimately yielding the ZIF‐8@FePt nanomaterials.

*PEG Modification on the Surface of ZIF‐8@FePt*: To improve colloidal stability in aqueous environments, ZIF‐8@FePt nanoparticles were surface‐modified with polyethylene glycol (PEG), which was selected for its exceptional solubility in water and substantial excluded volume.^[^ ^41^ ^]^ PEG chains were grafted onto the ZIF‐8 external surface using a diazonium‐induced anchoring process (DIAP), also called GraftFast, described by Gimenez‐Marques et al.^[^ ^42^ ^]^ 10 mg of PEG (average Mn 400) powder was dissolved in 100 mL PBS solution to prepare a 0.1 mg mL^−1^ PEG solution. Subsequently, 10 mL of this solution was added to the solution from step 3, followed by mechanical stirring (using a PTFE stirrer at 800 rpm) for 4 hours. Afterward, the mixture was centrifuged, and the supernatant was removed. PBS was added, and the solution was ultrasonically dispersed. This procedure was repeated five times. The mixture was centrifuged again, and 10 mL of PBS solution was added to obtain the final solution of MMP. Then, the solution of MMP in the VSM workspace with 1.8 T for magnetization was used to improve its locomotion performance in the magnetic field. 1 mL of this solution was taken out and dried to determine the mass, thereby calculating the concentration of MMP. Finally, the glue was dropped onto the dried surface of MMP, and then the magnetization profile of MMP was measured using the VSM.

### Characterization of MMP

*Powder XRD Measurements and Analysis*: Powder XRD characterization was carried out using a Stoe Stadi P diffractometer operating with Cu Kα_1_ radiation and a Ge(111) monochromator in Debye–Scherrer geometry. Samples were enclosed in 1.0 mm glass capillaries and rotated during acquisition to ensure representative particle statistics.

*Transmission Electron Microscopy and Scanning Electron Microscopy*: High‐resolution TEM imaging was performed on a Philips CM30 ST instrument operating at 300 kV with a LaB_6_ cathode. Specimens were deposited in dry form on copper lacey carbon grids (Plano), and micrographs were acquired using a TVIPS TemCam‐F216 CMOS detector. SEM investigations were carried out on Zeiss Merlin and VEGA TS 5130 MM (TESCAN) microscopes equipped with InLens detectors and operated at an accelerating voltage of 8 kV.

*Scanning Transmission Electron Microscopy and EDS*: High‐resolution imaging and analytical characterization—including HRTEM, electron diffraction, backscattered electron, and HAADF–EDS mapping—were performed using a JEOL ARM 200CF microscope operating at 200 kV. The system was configured with a field‐emission electron source, probe corrector, and dual silicon drift detectors for enhanced spatial and spectroscopic resolution.

*Zeta Potential Measurements*: The zeta potential was determined using a dynamic light scattering setup (Wyatt Mobius + Atlas Size & Zeta Potential Analyzer WMOB‐03 DLS). Dispersions of 0.1 mg mL^−1^ FePt, ZIF‐8, ZIF‐8@FePt, and MMP were separately prepared in PBS buffer solution (pH = 7.4) and sonicated for 15 minutes before the zeta potential measurements. Surface charge values are presented as the mean of three experiments, with the standard deviation indicated.

*Brunauer–Emmett–Teller Measurements*: For ZIF‐8, a material known for its high porosity and surface area, the Brunauer‐Emmett‐Teller (BET) test is a widely used method for measuring the specific surface area of materials based on the framework is particularly relevant. Nitrogen sorption analysis was carried out at 77 K using a Quantachrome Autosorb iQ MP system. Before measurement, all samples were degassed at 120 °C for 12 h under reduced pressure to remove adsorbed species. BET surface areas were obtained via multipoint fitting in the linear pressure region (0.05–0.1 P/P_0_), while pore size distributions were derived from the nitrogen adsorption isotherms using the NLDFT model for cylindrical pores in carbon.

*Radiofrequency (RF) Remote Heating of MMP*: The MMP was loaded in microtubes and irradiated with an RF heater (621.6 A, 338 kHz) at a 1 cm distance. Infrared thermography was performed using an ETS320 thermal imaging system (FLIR Systems, OR, USA) to record surface temperature distributions.

### Preparation of Polydimethylsiloxane (PDMS) Channels

The PDMS Y‐structure channel used for testing swarm behaviors of MMP in Figure 3A was fabricated from a 3D‐printed mold. The 3D mold is printed by a printer (Form 3B, Formlabs) using Clear v4 resin. PDMS prepolymer was prepared by mixing Sylgard 184 base and curing agent (Dow Europe) in a 10:1 volume ratio, followed by vacuum degassing. The mixture was cast over a patterned mold and degassed once more to eliminate trapped air. Curing was performed at 90 °C for 2 hours, yielding the completed PDMS‐based microchannel. A programmable pump (KD Scientific, 788270), operating at a constant flow rate of 2 mL min^−1^, was then used to inject the MMP solution into the Y‐shaped channel.

### Magnetic Field Control

A laser cutter (PLS6.150D, Universal Laser Systems, Inc.) was employed to engrave pre‐designed patterned channels on the acrylic surface for the patterned magnetic field. Neodymium‐iron‐boron (NdFeB) powder (5 µm) was mixed with PDMS in a 1:1 mass ratio. The mixture underwent degassing for 30 minutes to eliminate any trapped air before being poured into the patterned channels. Any excess mixture was then smoothed away using a lancet. The mold was placed on a hot plate and heated at 90 °C for 2 hours to allow the composite to cure. Finally, magnetization is performed in the VSM, with the magnetization direction perpendicular to the pattern plane.

For the experiments involving planar rolling, rotation, and upstream movement in channels of MMP, the MMP was controlled using a rotating permanent magnet sized 10 mm x 10 mm x 10 mm. The permanent magnet was embedded in a 3D‐printed PLA rotating base and mounted on a DC motor. The rotation speed of the DC motor was controlled by a programmed Arduino Uno Rev3.

The swarms of MMP were actuated using a custom‐built Helmholtz coil system comprising five coiled electromagnetic coils (4 xy‐coils and 1 z‐coil), as shown in Figure S6D (Supporting Information).

### Characterization of Drug Loading Degree and Efficiency

The drug loading capacity (DLC) and efficiency (DLE) of MMP were quantified indirectly, following the procedure outlined in the earlier studies.^[^ ^43^ ^]^ The supernatant collected after centrifugation of the final emulsion mixture (8 000 rpm, 1 h) used in the DOX‐ZIF‐8@FePt synthesis was analyzed using a microplate reader (Infinite 200 PRO, TECAN) at 485 nm. A calibration curve was constructed using a series of DOX standard solutions (1, 0.5, 0.25, 0.125, 0.0625, 0.03125, 0.0156, 0.0078, 0.0039, 0.00195, 0.000975, and 0.0000487) mg mL^−1^ (Figure S12A,B, Supporting Information). The MMP's DLC and DLE were calculated according to the following Equations (1) and (2):

(1) $$DLC\left(\%\right)=\frac{entrapped\;drug}{weight\;of\;MMP}\times 100\%$$

(2) $$DLE\left(\%\right)=\frac{total\;input\;of\;drug-amount\;of\;drug\;in\;the\;supernatant}{total\;input\;of\;drug}\times 100\%$$

### In Vitro Release of DOX

As an example drug for drug delivery experiments, doxorubicin, which is a chemotherapeutic drug against glioma and various other cancer types, is loaded into the MMP.^[^ ^43^ ^]^ The MMP dispersion was achieved by adding HCl to 1 mL of PBS containing 1 mL of MMP suspension with a known drug load, creating solutions of varying pH levels. The concentration of DOX in the supernatant was measured 24 hours later to evaluate the drug release rate. First, the absorption curve of DOX was detected at a concentration of 0.1 mg mL^−1^ in PBS buffer solution (pH = 7.4). Drug release behavior was examined by dispersing ZIF‐8@FePt nanoparticles in 1.0 mL of PBS buffer (pH 6.0 or 7.4) and incubating the mixture at 37 °C with gentle agitation in the dark. At specific time intervals, aliquots were collected by centrifugation at 16 000 rpm; 0.9 mL of the supernatant was analyzed using a microplate reader (Infinite 200 PRO, TECAN). The same volume of fresh PBS was replenished, and the suspension was sonicated to ensure complete nanoparticle dispersion before continuing incubation. The calibration curve for DOX quantification followed Y = 1.53846X + 0.03198 (R^2^ = 0.99812) (Figure S12B, Supporting Information), where Y indicates absorbance at 485 nm and X the DOX concentration (mg mL^−1^).

To explore the applicability of MMP within biological environments, their potential as drug carriers was investigated, focusing on DOX, a chemotherapeutic agent effective against multiple cancer types. The fluorescent properties of DOX enabled the quantification of its concentration by measuring the fluorescence intensity of the solution. Initially, seven aliquots of 200 µg DOX were each mixed into 1 mL of MEM containing 100 µg of MMP. The concentration of DOX in the supernatant was determined every three hours following centrifugation to calculate the loading efficiency. Approximately nine hours later, the loading efficiency of the MMP plateaued, reaching an efficiency of 93.9±2.65%, as depicted in Figure 4C.

### Culture of SH‐SY5Y and HBMEC Cell Lines

SH‐SY5Y neuroblastoma cells were maintained in DMEM/F12 (Gibco) supplemented with 10% fetal bovine serum and 1% penicillin–streptomycin at 37 °C in a 5% CO_2_ incubator. Media were renewed every 3–4 days. Before seeding, culture wells were coated with laminin (5 µg mL^−1^) in PBS containing Ca^2^⁺ and Mg^2^⁺ for 1 h at 37 °C. For viability assessment, cells were plated at 20 000 cells/cm^2^ in 96‐well plates and differentiated in DMEM/F12 supplemented with 1% FBS, 1% antibiotics, and 10 µm retinoic acid (Sigma‐Aldrich) for 4 days before experiments. Human brain microvascular endothelial cells (HBMECs) were cultured aseptically in EGM‐2 medium supplemented with 10% fetal bovine serum, 1% penicillin–streptomycin, and endothelial‐specific growth factors. Cultures were maintained at 37 °C in a humidified 5% CO_2_ atmosphere. To improve adhesion, type I collagen was used to pre‐coat culture flasks. The medium was changed every 2–3 days, and cells were enzymatically detached at 80–90% confluence with 0.05% trypsin–EDTA. Experiments were conducted with cells at passages 3–6 to preserve endothelial morphology and functionality.

### Biocompatibility Assays

To assess cytotoxicity, cells were treated with MMP suspensions (0, 5, or 10 µg mL^−1^) in their respective culture media. Viability was quantified after 24 and 48 hours using both CyQUANT LDH (Thermo Fisher Scientific) and CellTiter 96 Aqueous One MTS assays. Optical density was recorded with a BioTek Synergy 2 reader. Each treatment was performed in quadruplicate, and mean values from three independent biological replicates were used for statistical evaluation.

### J774A Macrophage Cell Culture and Flow Cytometry

J774A.1 murine macrophages were cultured in DMEM supplemented with 10% fetal bovine serum and 1% penicillin‐streptomycin at 37 °C in a 5% CO_2_ humidified incubator until treatments. The F4/80 and CD11b antibody staining for cell surface markers was performed on two groups: Untreated and MMP‐treated J774A macrophages. The final concentration of 10 µg mL^−1^ MMP was added into the solution in the T75 flask containing J774A cells at passage number 11. Cells were collected after 24 or 48 hours of incubation, centrifuged, and incubated with antibodies at 4 °C for 30 minutes in the dark. Following three washing steps, the cells were suspended in staining buffer and analyzed by flow cytometry (BD LSRFortessa X‐20, BD Biosciences). For each condition, at least 10 000 events were acquired, and data analysis was performed using FlowJo software (version 10.8.1).

### Imaging of Tissue Mimicking Phantoms

issue‐mimicking phantoms (2 cm diameter) were prepared from 1.5% agar and 0.4% Intralipid (w/v) in distilled water. Cavities were molded to hold clear straws containing magnetic nanoparticle suspensions of defined concentrations, which were embedded into the phantom structure. Imaging was carried out using a Multispectral Optoacoustic Tomography system (InVision 256‐TF, iThera Medical, Munich, Germany). Optoacoustic signals were recorded across 56 wavelengths (550–1100 nm, 10 nm steps) at 25 °C, with three frames per wavelength averaged to enhance signal quality.

### Imaging of MMP in Ex Vivo Porcine Brain Model

Porcine organs and whole blood were obtained from a local abattoir, stored at 5 °C, and utilized within 24 h of collection. The brain was specifically employed to evaluate MSOT imaging performance. Tissues were used as received, without additional washing. Sections containing both major and collateral vessels were excised with a scalpel to allow controlled blood outflow. For circulation through the phantom system, a needle was cannulated into a primary vascular branch. A solution of MMP (1 mg mL^−1^ in phosphate‐buffered saline, PBS) was perfused into the cerebral vasculature at a flow rate of 1 µL s^−1^ using an external syringe pump. The specimen was positioned on an agar support within the imaging field of the MSOT probe, which was oriented upward to permit magnetic nanoparticle (MNP) manipulation via a handheld rotating magnet. To reduce image artifacts, a custom‐designed blood retainer was applied to limit blood pooling in the imaging region.

### In Vivo Mouse Imaging Experiments

All animal experiments were approved by the Cantonal Veterinary Office Zürich (Approval Numbers: ZH092/2022 and ZH060/2022) and conducted in accordance with the Swiss Federal Act on Animal Protection. The animals were kept under ad libitum conditions at a temperature of 22 ± 2 °C and under a 12‐hour light/dark cycle until the start of the experimental procedures. In vivo multispectral optoacoustic tomography (MSOT) was carried out using three female athymic nude mice (8 weeks old, ≈22 g). Mice were anesthetized with isoflurane (5% induction, 1.5% maintenance; Abbott, Cham, Switzerland) in an oxygen/air mixture (100/400 mL min^−1^) and housed under standard conditions (21 ± 1 °C, 55 ± 10% humidity, 12 h light/dark cycle) with unrestricted access to food and water. For imaging, the eyes were protected with dexpanthenol ointment, and the head was stabilized in a stereotactic frame. The animals were placed prone on a temperature‐controlled heating pad (37 °C) lined with soft material. MMPs (1 mg mL^−1^, 1 mL total) were administered intravenously through the tail vein or femoral artery. Imaging was conducted using a custom‐built volumetric MSOT device comprising a 512‐element spherical transducer array (7 MHz, Imasonic SaS, Voray, France) and illuminated by an 800 nm pulsed laser (100 Hz repetition rate; Spitlight EVO‐III, Innolas Laser GmbH, Krailling, Germany) coupled via a 3 mm liquid light guide (NA ≈ 0.55). Data were recorded for 5 min (30 000 volumetric frames): 30 s pre‐injection, 30 s during, and 4 min post‐injection.^[^ ^44^, ^45^ ^]^

### Real‐Time In Vivo MSOT Imaging of Magnetically Directed Intravascular Motion

To assess the in vivo magnetic manipulation of the synthesized particles, the mouse was placed in a supine orientation above the downward‐facing 512‐element spherical array transducer, with the imaging chamber filled with deionized water. The brain was acoustically coupled to the imaging system using ultrasound gel. Optoacoustic images were captured at a repetition rate of 100 Hz and an excitation wavelength of 800 nm for 4 minutes (24 000 frames). A rotating NdFeB electromagnet (20 × 20 × 20 mm) was positioned over the brain and activated 1 minute after administration of MMPs.

### Localization Optoacoustic Tomography (LOT)

LOT images were reconstructed from the volumetric time‐lapse MSOT datasets according to the procedure described elsewhere.^[^ ^44^ ^]^ In brief, the raw data were first processed with a bandpass filter spanning 0.1–7 MHz. To suppress tissue‐related clutter and residual high‐frequency noise, a singular value decomposition (SVD) filter was applied. Specifically, Casorati matrices were constructed from data blocks of size (493 × 512 × 200), and eigenvectors within the range of 25 130 were retained to capture signals associated with rapidly moving microparticles. Following this, local intensity peaks were identified in the filtered data, and particle trajectories were reconstructed across successive frames using a tracking algorithm (simpletracker.m, MathWorks; based on the Matlab Munkres algorithm by Yi Cao 2009, wrapped by Jean‐Yves Tinevez 2019).^[^ ^45^ ^]^

### MR Imaging of MMP

MRI measurements were acquired on a 7 T Bruker BioSpec scanner (Ettlingen, Germany) equipped with an actively shielded BGA20SHP gradient system and a 40 mm quadrature birdcage coil. Excised porcine cerebellum samples (N = 3) were imaged both before and after injection of MMP suspension (20 µL, 1 mg mL^−1^) using two pulse sequences: a 2D gradient‐echo protocol (TR/TE = 500/7 ms, 113 × 113 µm^2^ resolution, 0.5 mm slice, NEX = 4, 10 min total) and a RARE spin‐echo method (TR/TE_eff = 3121/40.7 ms, RARE factor = 8, same resolution, NEX = 4, 7 min 32 s duration).

### Statistical Analysis

All experiments were repeated independently at least three times for replicability. The GraphPad Prism 10 software was used for the statistical analyses. Two experimental groups were tested with a *t*‐test, multiple experimental groups with one‐way ANOVA, and multiple experimental groups with several time points were statistically tested with two‐way ANOVA. Quantitative data in figures were presented as mean ± standard deviation (SD).

## Conflict of Interest

The authors declare no conflict of interest.

## Author Contributions

F.W. and M.S. proposed the research. F.W. and M.S. designed the study with help from all authors. F.W. designed the fabrication protocol. F.W. and E.Y., assisted by Y.Y., S. L., D.S., and X.L.D., designed and performed the biological, ex vivo, and in vivo demonstrations. J.Z. helped with the MRI imaging experiment. F.W., assisted by W.K., S.R.H., and S.Z., designed and drew the figures. F.W. wrote the manuscript. M.S., D.R., and E.Y. supervised the research. All authors analyzed the data and discussed the results. All authors edited and commented on the manuscript.

## Supporting information



Supporting Information

Supporting Information

Supporting Information

Supporting Information

Supporting Information

Supporting Information

Supporting Information

Supporting Information

Supporting Information

Supporting Information

Supporting Information

Supporting Information

## Data Availability

The data that support the findings of this study are available on request from the corresponding author. The data are not publicly available due to privacy or ethical restrictions.
