# Supplementary material for: Optoacoustic‐Guided Magnetic Microrobot Platform for Precision Drug Delivery
Source: Adv Mater. 2025 Oct 25;38(5):e11870. doi: 10.1002/adma.202511870 (PMC12822537; doi:10.1002/adma.202511870)
Supplement: Supplementary file 1 — Supporting Information [file ADMA-38-e11870-s012.pdf]

ADVANCED MATERIALS

Supporting Information

for *Adv. Mater.*, DOI 10.1002/adma.202511870

Optoacoustic-Guided Magnetic Microrobot Platform for Precision Drug Delivery

Fan Wang, Erdost Yildiz, Xosé Luís Deán-Ben, Yan Yu, Daniil Nozdriukhin, Wenbin Kang, Shuaizhong Zhang, Jelena Zinnanti, Devin Sheehan, Ren Hao Soon, Shuxin Lyu, Daniel Razansky* and Metin Sitti**

Supplementary Materials for

Optoacoustic-Guided Magnetic Microrobot Platform for Precision Drug Delivery

The PDF file includes:

Note S1: Magnetic Actuation Methods for Nanoparticles

Note S2: MOF Material Properties

Note S3: Magnetic Heating Mechanism

Note S4: Locomotion Mechanism

Note S5: Comparison of Medical Imaging Technologies for Real-time Visualization and Guidance of Micro-/Nanorobotics

Figures S1-S16

Tables S1-S3

Legends for Movies S1-S11

Note S1: Magnetic Actuation Methods for Nanoparticles

To power and control nanoparticles for effective drug delivery to specific locations, a variety of mobile nanoparticles have been developed utilizing different actuation methods, including magnetic [1,2], optical [3,4], acoustic [5,6], electric [7], and chemical approaches [8,9] (**Table S1**). Usually, magnetic nanoparticles used as micro/nanorobotics (MNRs), driven by rotating magnetic fields (RMFs) to generate forward thrust, have become one of the most extensively studied due to the ease of remote control within flowing blood vessels using magnetic fields[10-12]. The choice of magnetic material is critical in limiting the motion of MNRs against strong fluid flows. Commercially available magnetic materials like iron, nickel, and cobalt have low remanence, making them less effective for actuation by weak uniform magnetic fields, and the biocompatibility of nickel- and cobalt-based materials is highly contentious[13]. The $L1_0$ phase of iron-platinum (FePt) is a promising hard-magnetic material for micro-/nanorobotics, offering high remanence, coercivity, biocompatibility, and stability. Its integration into MNRs allows for leveraging its unique advantages, such as high remanence for better actuation in weak rotating magnetic fields, enabling navigation at higher flow velocities, and biocompatibility for safer use within the human body [14-16]. Moreover, due to the unique magnetic and electromagnetic properties of FePt, when FePt particles are exposed to a radio frequency (RF) magnetic field, they can absorb the RF energy and convert it into thermal energy[17]. However, it is hard to accomplish a therapeutic task alone with a single MNR; envisioned are swarms of microrobots that deliver therapeutic payloads to tissues, controlled externally by magnetic fields to reach target locations in reconfigurable collective forms. A sufficient number of MNRs not only enhances propulsion but also allows for tracking by conventional medical imaging techniques due to their collective quantity[18]. This approach underscores the potential of utilizing advanced magnetic materials and swarm behavior for precise, controlled delivery of therapeutics, highlighting the need for further innovation in MNR design and application for minimally invasive medical treatments.

Note S2: MOF Material Properties

On top of the propulsion strategies of nanoparticles, however, mobile nanoparticles, such as MNRs, also need to be designed to carry a sufficient amount of medication and precisely control the release of pharmaceutical agents to achieve therapeutic effects. Current research predominantly focuses on propulsion strategies, with relatively less exploration into enhancing drug loading capacities[19]. Some

studies attempt drug carriage via electrostatic interactions on the particle surface, but this method offers limited drug loading, lower stability, and is prone to drug leakage and uncontrolled release [20]. However, metal-organic frameworks (MOFs), as porous materials with high porosity (pore size from around 8-55 Å), large surface area (from 1300 m^2 g−1 to 4000 m^2 g−1), and tunable functionalities, are considered potential candidates for drug encapsulation and carriers in biomedical applications due to their defined pore structure[21-23]. Zeolite imidazolate frameworks (ZIF) are a subclass of MOFs currently used in gas separation, chemical separation, and as carriers for metal nanoparticles and drugs. Zeolitic imidazolate framework-8 (ZIF-8), composed of zinc ions and 2-methylimidazole (2-MI), stands out as one of the most promising MOFs for its non-toxicity and excellent biocompatibility[24,25]. ZIF-8 nanoparticles are stable under physiological conditions and can decompose in acidic environments such as tumor tissues and acidic organelles due to the protonation effect, making them highly suitable for constructing pH-responsive drug delivery systems[26-28]. Additionally, ZIF materials have been employed to encapsulate small molecule drugs, macromolecular drugs, enzymes, and even nanoparticles (NPs), including various precious metal NPs (such as Pt, Pd, and Au), for the development of core/shell nanocomposites with novel functionalities and biomedical applications[29].

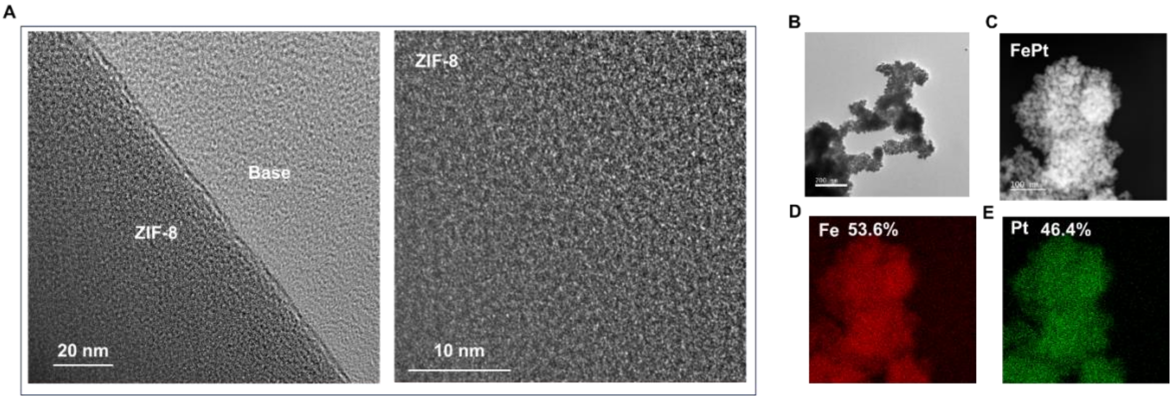

Figure S1: (A) TEM images of ZIF-8. **(B)** TEM image of FePt nanoparticles. **(C-E)** STEM and EDS images of FePt and Fe, Pt mapping.

Note S3: Magnetic Heating Mechanism

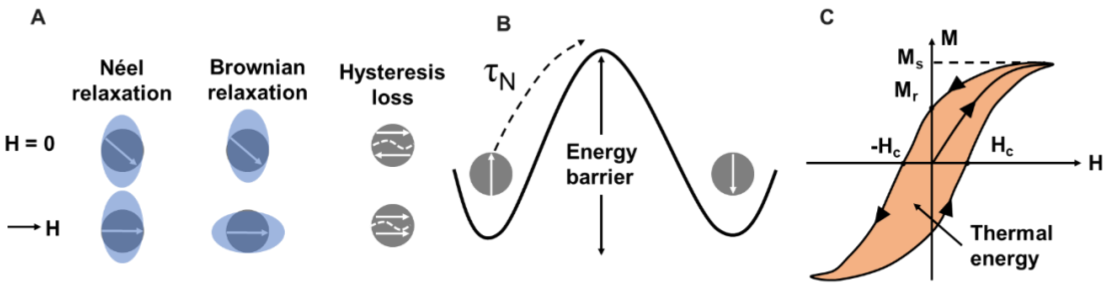

Figure S2: (A) Magnetic heating mechanism. **(B)** Energy barrier governing single-domain particles using the Néel relaxation model. **(C)** Representation of heat generated via hysteresis losses.

As shown in **Fig. S2A**, the magnetic nanoparticles can release heat under a high-frequency alternating magnetic field (AMF) via three distinct mechanisms: Néel relaxation (rotation of internal magnetic moment), Brownian relaxation (physical rotation of the magnetic nanoparticles), and hysteresis losses (rotation of magnetic domains) [37].

As FePt NPs with coating ZIF-8 were used in this research, the FePt NP can be considered as single-domain magnetic nanoparticles. The heat dissipation via Néel relaxation is caused by the internal rotation of the magnetic moment of the magnetic NPs at high-frequency AMF. The magnetic moment rotates over the anisotropy energy barrier $E = KV_c$, as depicted in **Fig. S2B**, and the Néel relaxation time τ_N can be expressed as follows [38]:

$$\tau_N = \tau_0 \exp\left(\frac{KV_m}{k_B T}\right) \quad (1)$$

where $\tau_0=10^{-9}$ s, K denotes the anisotropy constant, V_m represents the volume of magnetic nanoparticles, k_B denotes the Boltzmann constant, and T indicates the temperature. At low-frequency AMF ($1/f \ll \tau_N$), the magnetization of magnetic NPs changes instantaneously with the applied field; however, at high frequencies ($1/f$ close to τ_N), the magnetization lags from the applied magnetic field and causes magnetic hysteresis. In particular, the amount of thermal energy generated during a magnetization cycle can be expressed as follows [39]:

$$\mathrm{E} = \int_{-H_{max}}^{+H_{max}} \mu_0 M(H) dH \quad (2)$$

where μ_0 denotes the permeability of free space, and M(H) represents the magnetization of the magnetic nanoparticles. Therefore, the energy dissipated equals the area of the hysteresis loop, as portrayed in **Fig. S2C**.

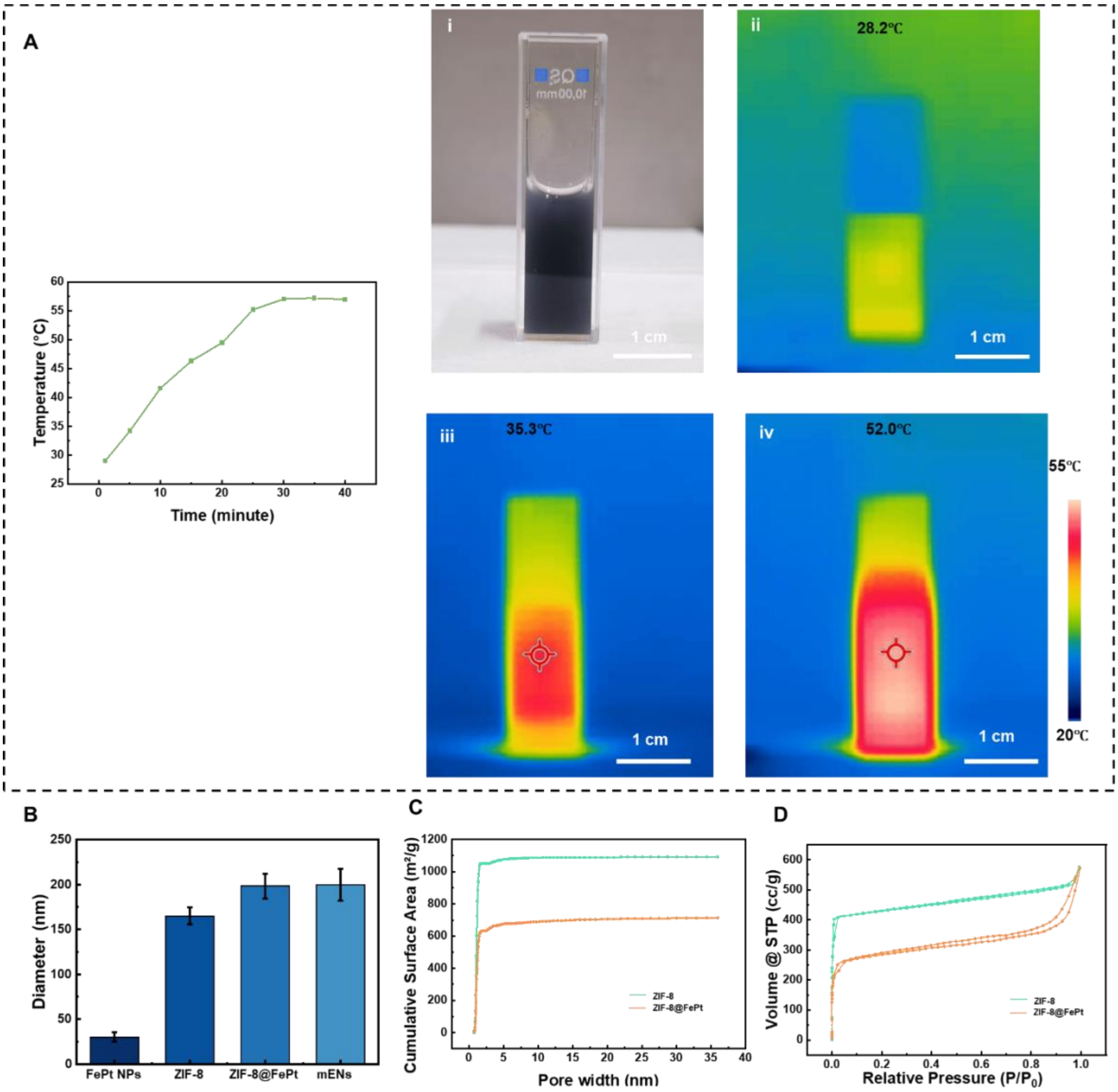

Figure S3: (A) Temperature changes with RF heating. **(B)** Average diameter of FePt NPs, ZIF-8, ZIF-8@FePt, and mPNs. **(C)** Cumulative surface area of ZIF-8@FePt and ZIF-8. **(D)** Nitrogen adsorption/desorption isotherms for ZIF-8 and ZIF-8@FePt, providing insight into their surface area and porosity characteristics.

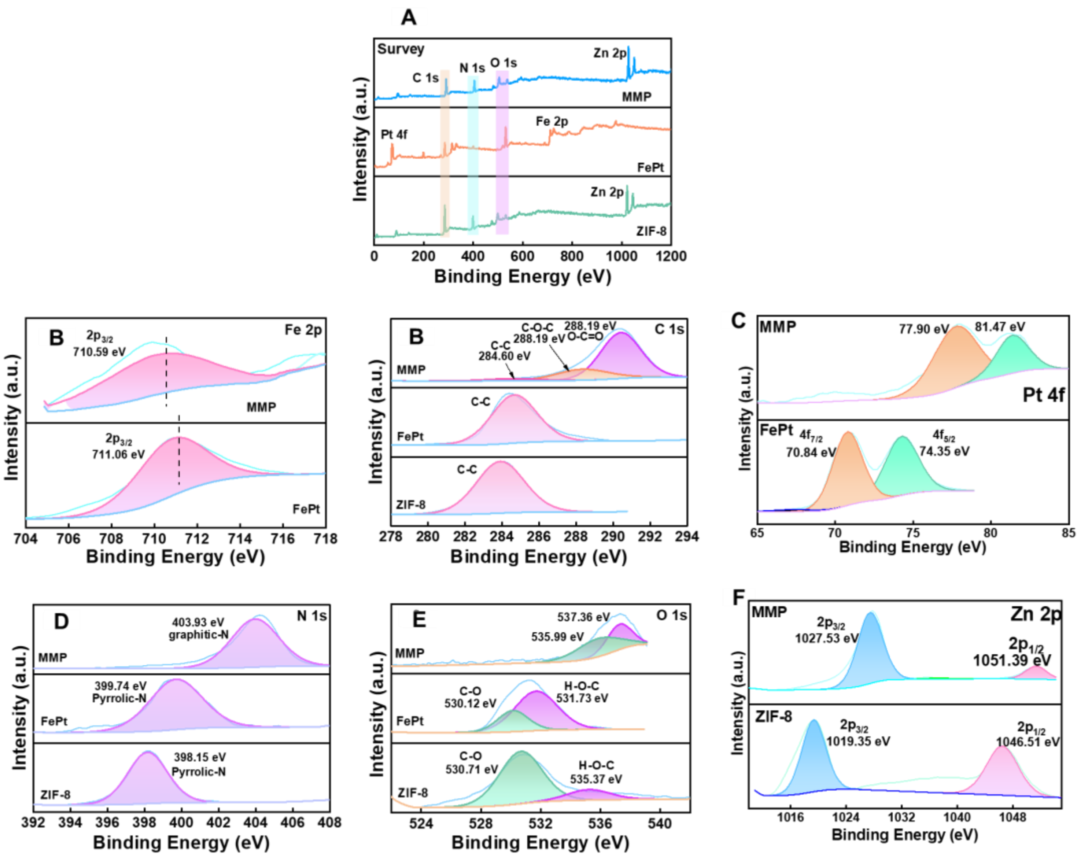

Figure S4: X-ray Photoelectron Spectroscopy (XPS) analysis. a.u., arbitrary units.

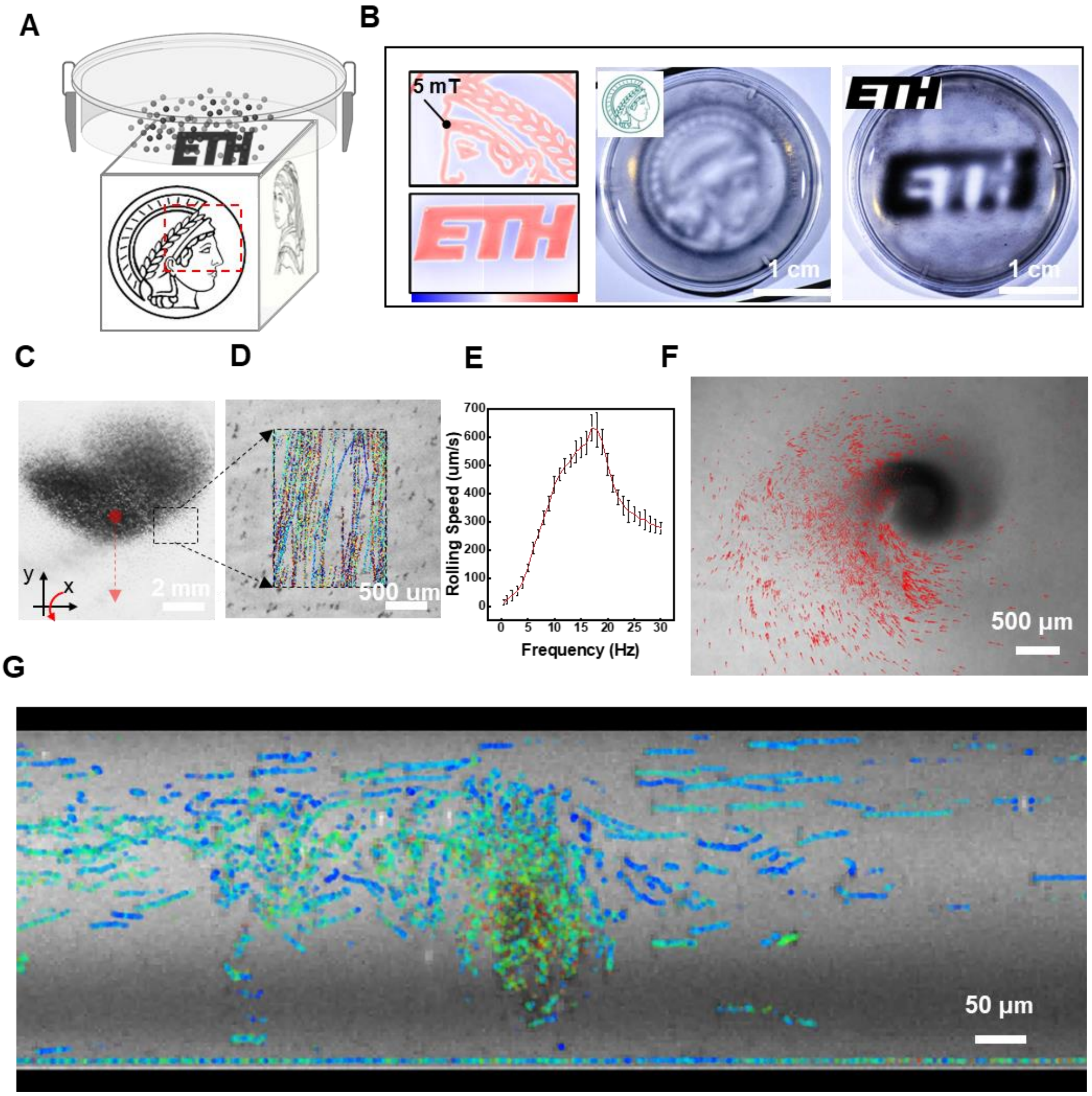

Figure S5: (A) Experimental setup of pattern magnetic field. **(B)** Details of Magnetic flux density measurements of magnetically patterned fields (the head of the Minerva). The maximum magnetic value is around 5 mT. (**C**) The MMP exhibits flock-like collective behavior under the control of a rotating magnetic field. (**D**) The rolling behavior, instantaneous velocity, and movement trajectory of the MMP under magnification through a microscope. (**E**) The variation in the rolling speed of the MMP under magnetic fields with different rotation frequencies. **(F)** Vortex behaviors of the MMP. **(G)** The MMP locomotion and tracing upstream in the tube.

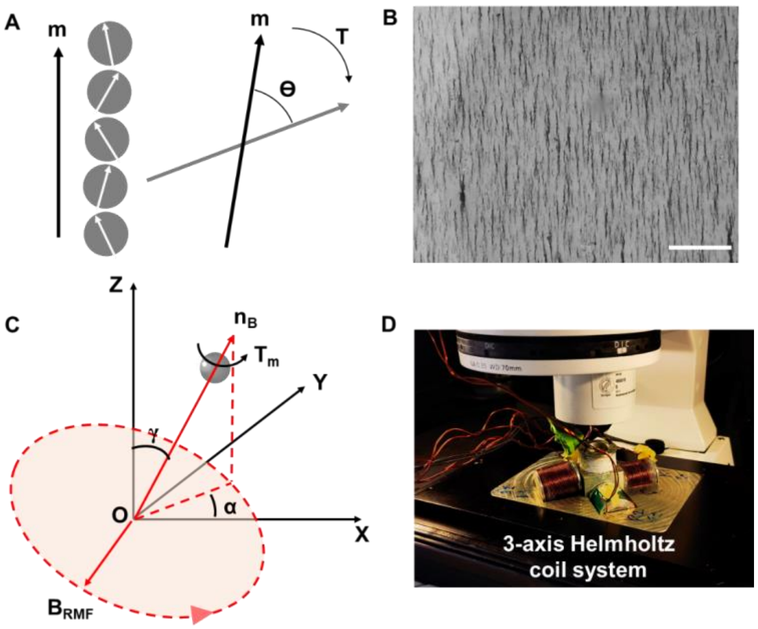

Figure S6: Locomotion mechanism. (A, B) Demonstration of aligned magnetic nanoparticle chains utilizing magnetic torque for active locomotion. **(C)** Illustration of a uniform rotating magnetic field and the control method based on a spring mechanism. **(D)** Three-axis Helmholtz coil system for active locomotion.

Note S4: Locomotion Mechanism

In particular, the net magnetic torque experienced by a randomly oriented MMP is approximately zero. However, magnetically aligned, the MMP experiences a stronger net magnetic torque that is maximum for a magnetic field applied in their perpendicular direction, as depicted in **Fig. S6A**. Therefore, the fabricated magnetically aligned spring synchronizes with the applied uniform rotating magnetic field and produces a rotational motion along the long axis of the line, which induces a propulsive force along the same axis (**Fig. S6B**).

The active locomotion of the MMP depends on the rotation caused by the magnetic torque, which can be expressed as follows [40]:

$$T_m = VM \times B_{ext} \quad (3)$$

where V denotes the volume of the MMP, M indicates the magnetization, and Bext represents the external magnetic field density. In addition, the 3D locomotion control of the MMP is summarized in **Fig. S6C** using a rotating magnetic field. If a uniform rotating magnetic field (BRMF) is applied to the MMP, the magnetic moment of the MMP synchronizes with the applied field, and a propulsive force is generated along its long axis. As the propulsion direction of the MMP is identical to the axis of the BRMF rotation (NB), the locomotion direction can be controlled by adjusting the orientation of NB via the control of the azimuthal (γ) and polar (α) angles. Therefore, NB can be expressed as

$$N_B = [\sin(\gamma)\cos(\alpha), sin(\gamma)\sin(\alpha), \cos(\gamma)]^T \quad (4)$$

and $\boldsymbol{B}_{RMF}$ can be expressed as

$$B_{RMF} = \begin{bmatrix} B_x \\ B_y \\ B_z \end{bmatrix} = B_0 \begin{matrix} \cos(\gamma)\cos(\alpha)\sin(\omega t) + \sin(\alpha)\cos(\omega t) \\ [\cos(\gamma)\sin(\alpha)\sin(\omega t) - \cos(\alpha)\cos(\omega t)] \\ -\sin(\gamma)\sin(\omega t) \end{matrix} \quad (5)$$

Moreover, the three-axis Helmholtz coil system used for locomotion control is presented in **Fig. S6D**.

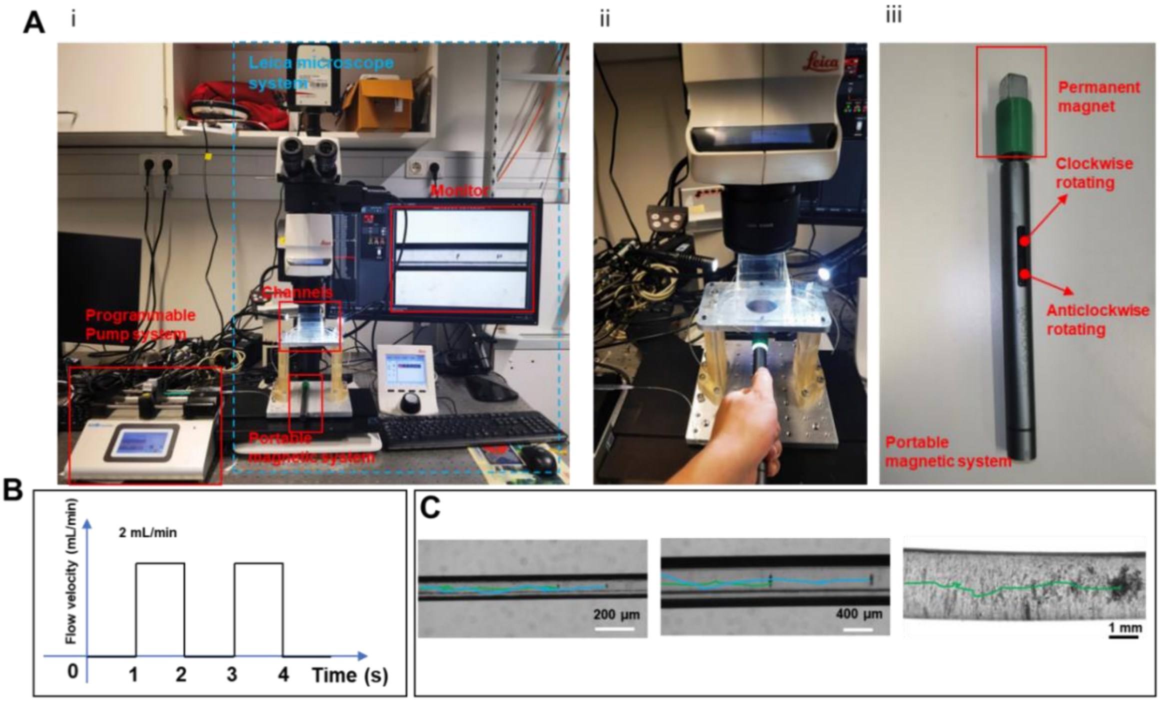

Figure S7: (A) (i) Experimental setup under a rotating magnetic field. **(ii)** Transparent microfluidic channel for pulsatile flow experiments. **(iii)** Portable rotating magnetic system with clockwise and anticlockwise rotating buttons. **(B)** Pulsatile flow controlled by the programmable pump system. **(C)** MMPs Upstreaming under various-sized tubes in Pulsatile flow.

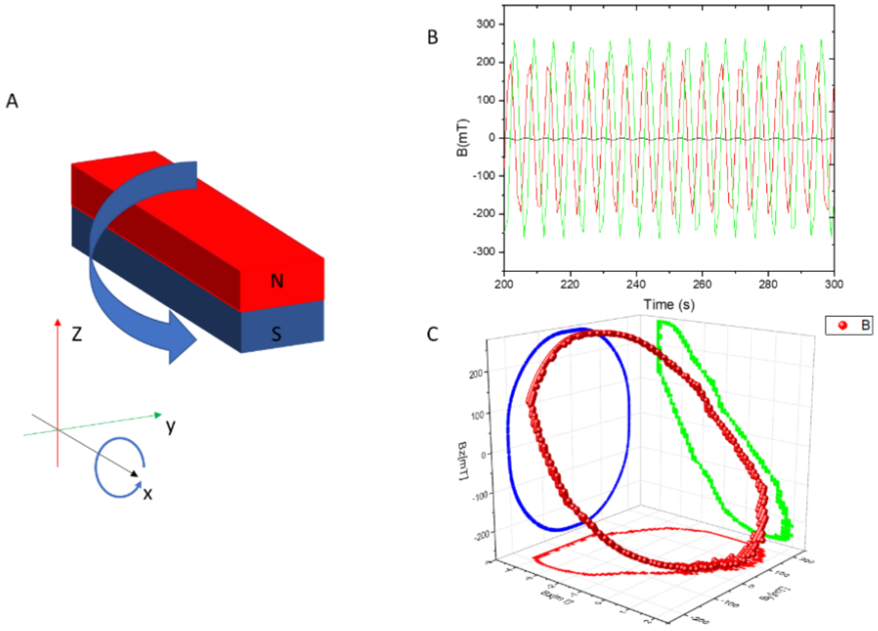

Figure S8: Rotating permanent magnet features. (A) Schematic diagram of a rotating permanent magnet device. **(B)** Magnetic Field Distribution on the X, Y, and Z Directions (2D Plots). **(C)** Magnetic Field project on the X-Y, Y-Z, and X-Z planes (3D Representation).

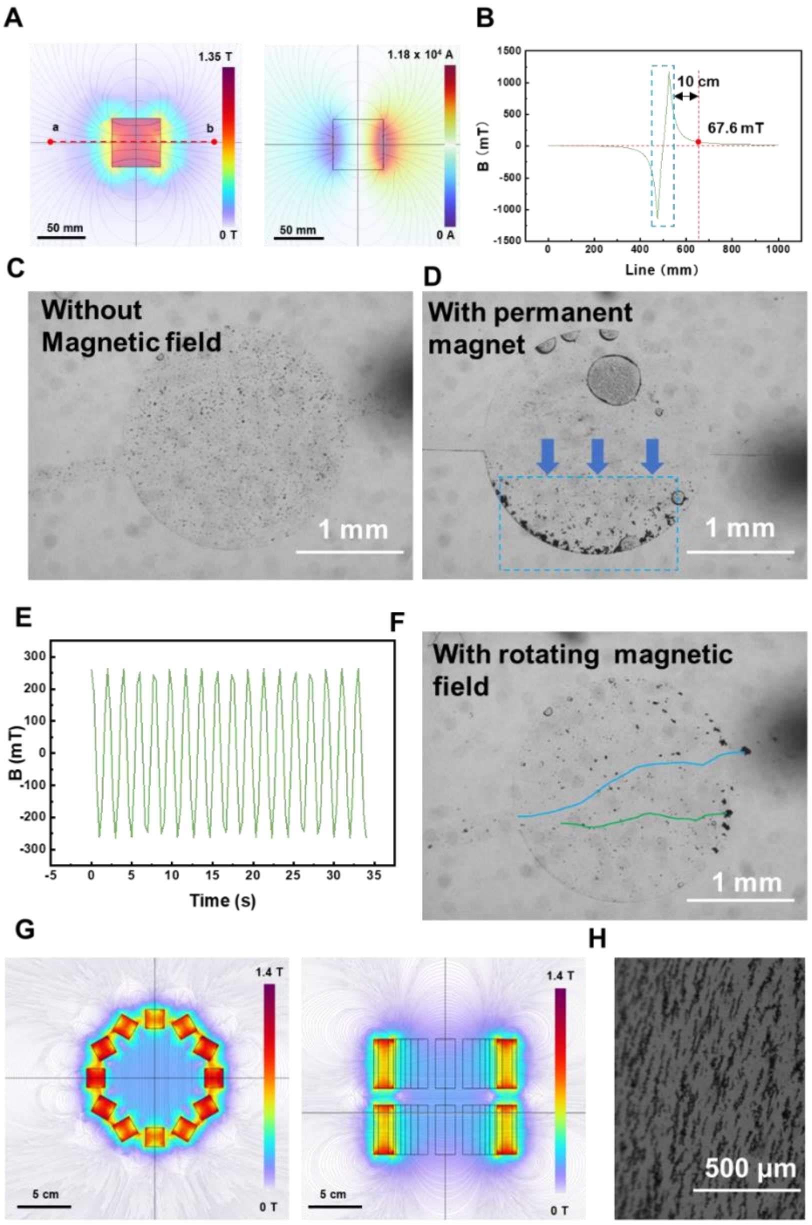

Figure S9: Comparison of magnetic field configurations for MMP manipulation. (A) Simulated magnetic field distribution of a permanent magnet (5 cm × 5 cm × 5 cm) and **(B)** the field profile along the line from point a to point b. **(C–D)** MMP distribution in a microchannel without **(C)** and with **(D)** a permanent magnet**. (E)** Magnetic field variation of a rotating permanent magnet measured 1 cm from its surface, and **(F)** the corresponding MMP trajectory under the rotating field. **(G)** Simulated magnetic field distribution of a Halbach array (inner diameter: 10 cm) and (**H**) the MMP distribution at its center.

Note S5: Comparison of Medical Imaging Technologies for Real-time Visualization and Guidance of Micro-/Nanorobotics

Although rapid advancements in micro/nanorobotic research, medical imaging, and wireless control of MNRs within the body remain significant challenges in practical medical research. [30] There is an urgent need for real-time visualization and guidance of MNRs on a whole-body scale with high spatiotemporal resolution to observe the dynamics of MNRs within the body directly. [31] Beyond high spatiotemporal resolution, an ideal non-invasive imaging technique should offer deep tissue penetration and molecular contrast. Optical imaging has been extensively applied in the biomedical field for its high spatiotemporal resolution and molecular contrast. However, its application to deep tissue is hindered by significant optical scattering, limiting high-resolution imaging beyond the optical diffusion limit (approximately 1 to 2 mm in depth)[32]. While MRI and CT provide unparalleled depth and resolution in imaging, enabling advanced tracking of micro- and nanorobots, these modalities have substantial challenges, including high maintenance expenses and a lack of portability. Furthermore, the powerful magnetic fields inherent to MRI and the significant radiation exposure from CT complicate the operational control of micro and nanorobots and constrain material selection. Thus, identifying and developing alternative imaging techniques is imperative for the continued innovation and application of micro and nanorobotics in medical fields. Fortunately, optoacoustic tomography (OAT) achieves high-resolution imaging far beyond the optical diffusion limit by detecting photon-induced ultrasound waves[33]. In OAT, the energy absorbed by chromophores within tissues is converted into sound waves, which are then detected to generate high-resolution tomographic images with optical contrast. Operating within the near-infrared (NIR) wavelength range, OAT enables completely non-invasive imaging of the entire mouse brain (with intact skull and scalp) with high contrast and high spatiotemporal resolution. Notably, OAT allows imaging at the scale of whole mouse organs with a three-dimensional spatial resolution as low as 100 μm and a volumetric rate of 100 Hz, making real-time 3D tracking and control of MNRs in an actual biological environment feasible[34-36]. With these advantages, OAT brings hope for real-time navigation of in-body micromotors, with broad applications in fields such as drug delivery.

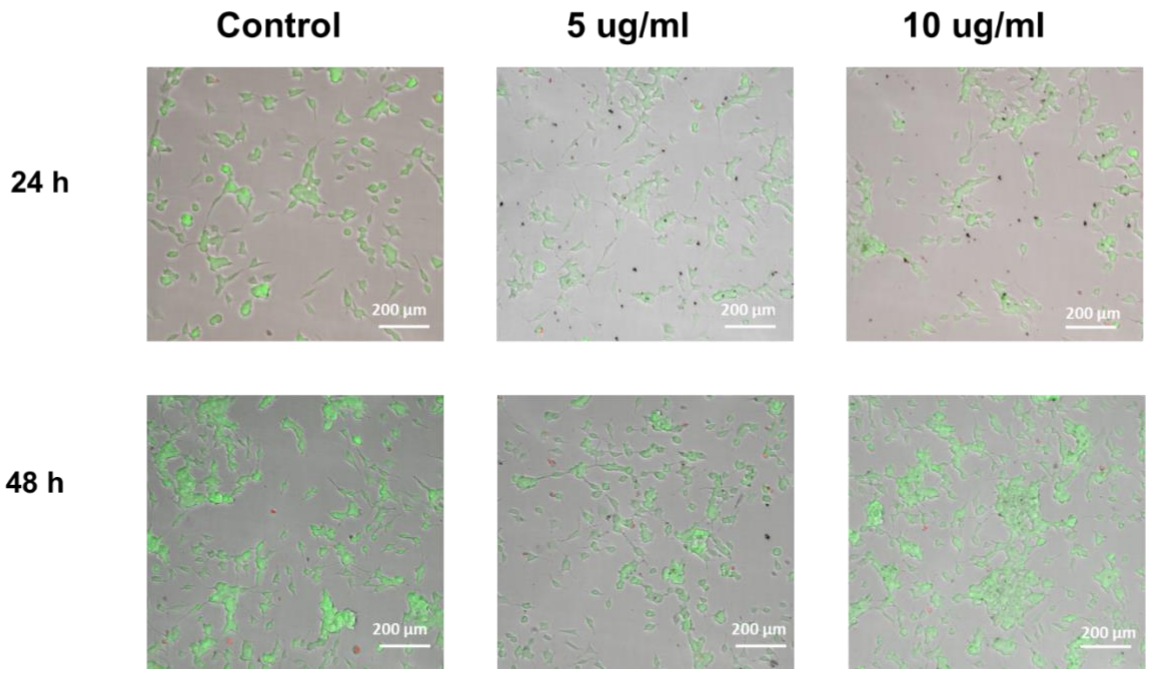

Figure S10: Overlapped images of brightfield and fluorescence microscopy images of SH-SY5Y cells cultured with the MMP for biocompatibility testing at 24 and 48 hours. (While green-stained cells indicate alive cells, red-stained cells indicate dead cells.)

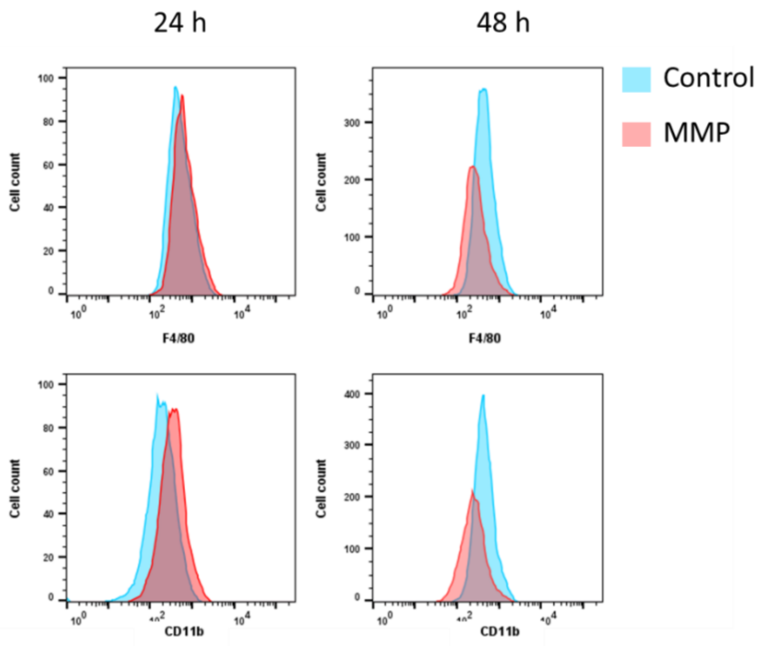

Figure S11: Individual counts of flow cytometry results for the F4/80 and CD11b expression on macrophages with and without MMP.

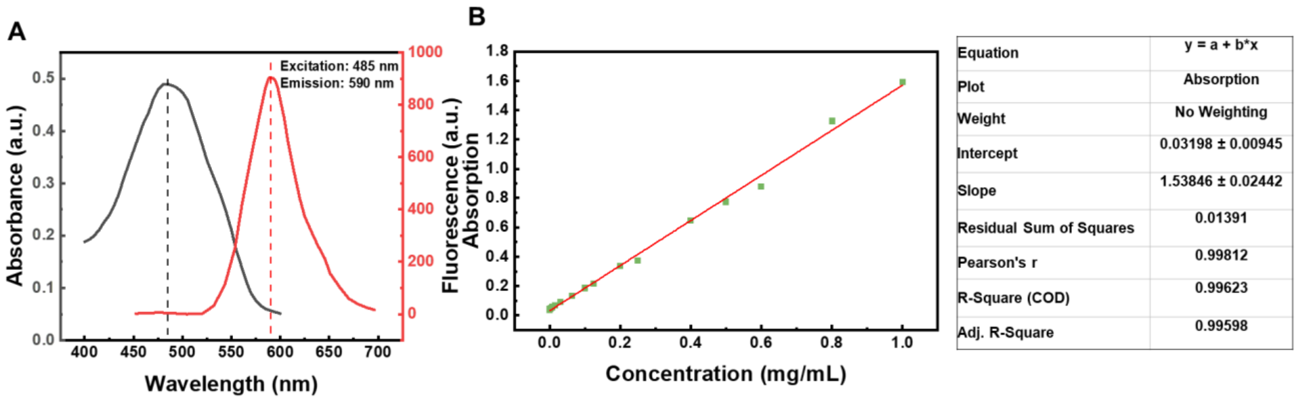

Figure S12: (A) Absorbance and fluorescence curve of DOX at a concentration of 0.1 mg/ml in PBS buffer solution (pH = 7.4) for spectrophotometric measurements. **(B)** Standard calibration curve of known DOX concentrations.

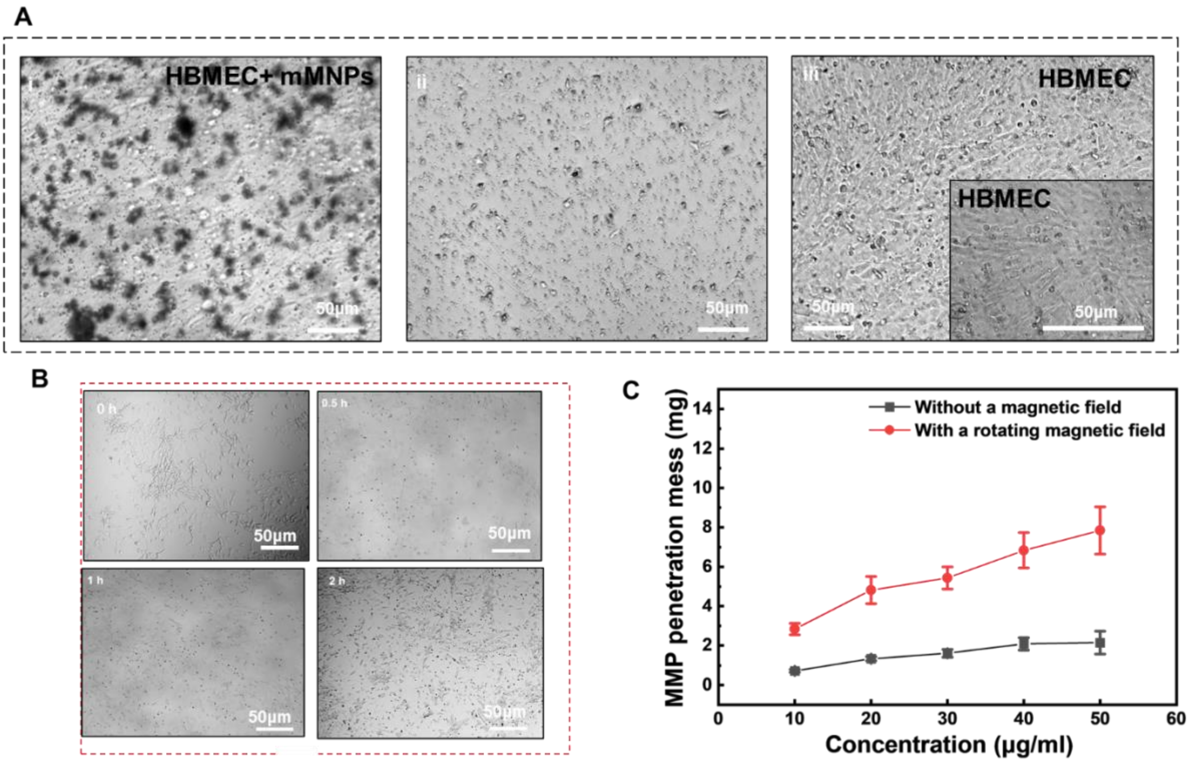

Figure S13: (A) HBMEC cells cultured with the MMP on the transwell. **(B)** The MMP transfers through the BBB model to the chamber after rotating magnetic field actuation. **(C)** Comparison of the penetration capacity of the MMP across an artificial blood-brain barrier with and without a rotating magnetic field.

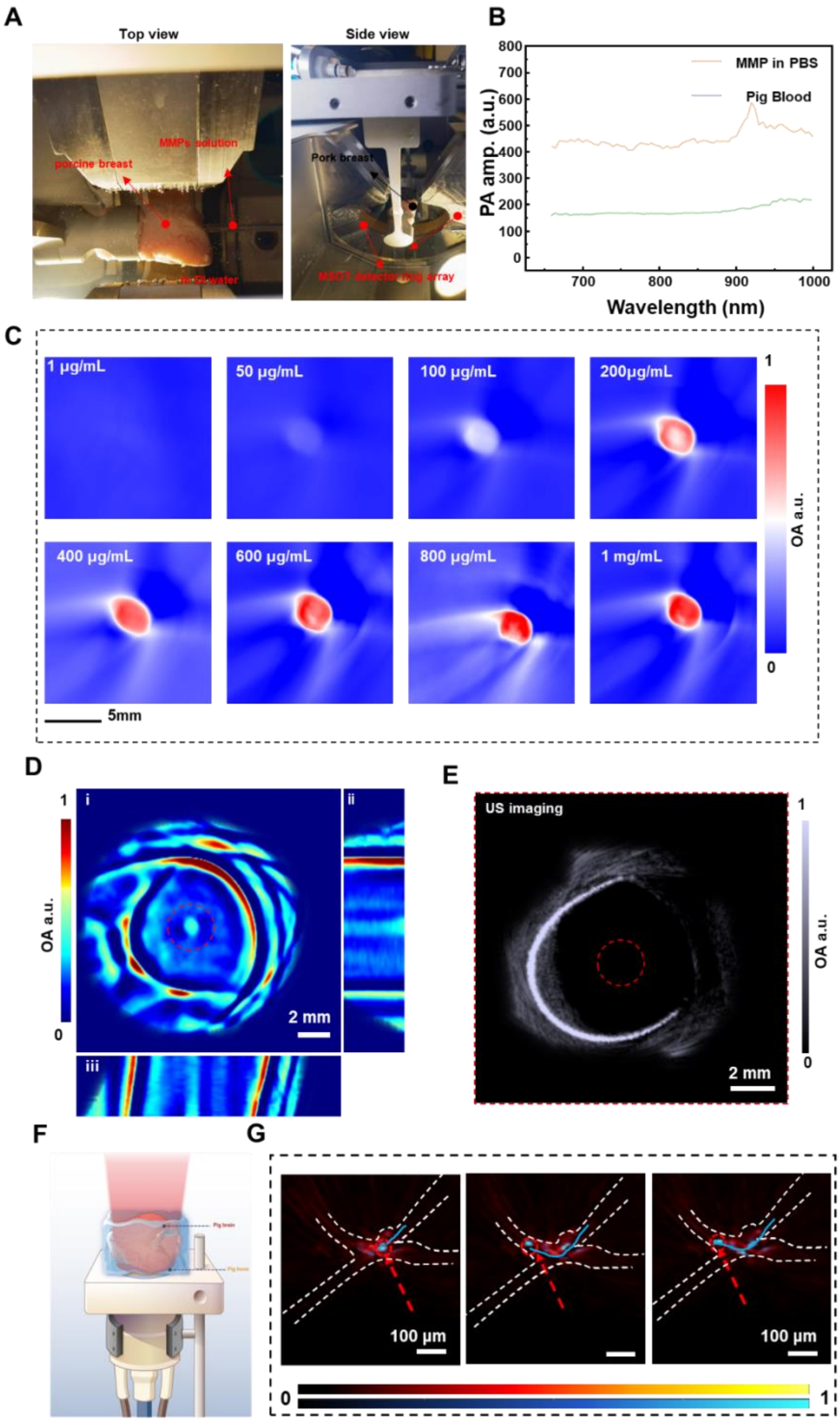

Figure S14: MSOT imaging of particles in various ex vivo organs. (A) MSOT imaging system with ring detectors in the DI water tank. **(B)** Optoacoustic spectrum analysis comparing the MMP and pig blood. **(C)** The corresponding OA images of the MMP with different concentrations. **(D)** OA images of the MMP around a bone tube. **(E)** Ultrasound image of the MMP around a bone tube. **(F)** Schematic description of the MMP injection procedure into the pig brain surface vessel. **(G)** OAT images of tracking the MMP in the brain vessel.

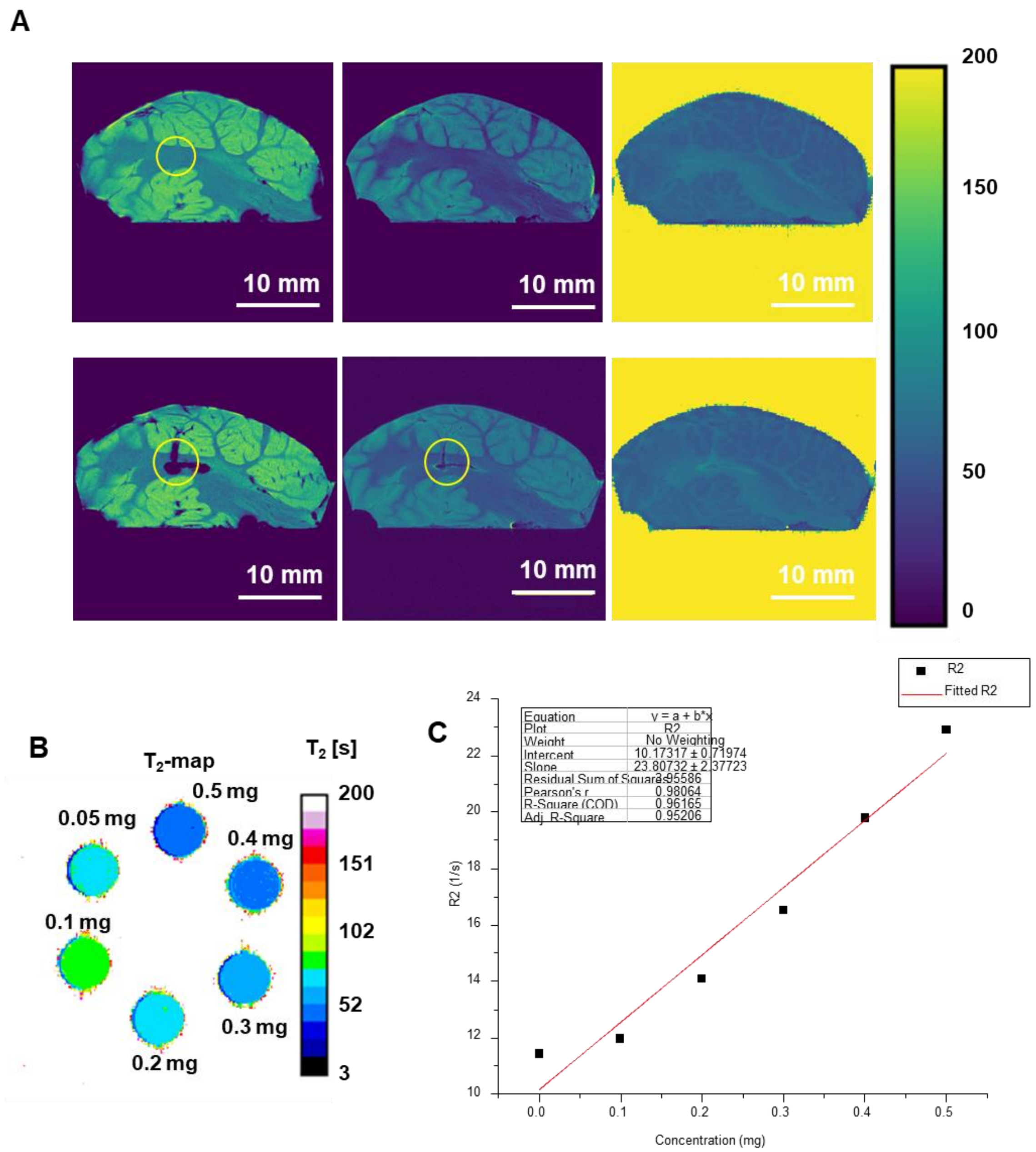

Figure S15: MRI imaging of MMP in the pig brain tissue. (A) MRI images of the MMP injection into the pig brain surface vessel. **(B)** Sets of T2-weighted images from different MMP aqueous solutions. The concentrations in the maps are solely based on the magnetic MMP (mg/ml), and the MMP was suspended in methanol with 0.5% agarose. **(C)** Transverse relaxation rates (R_2 = 1/T2) as a function of the concentration of the MMP for 7 T. Transverse relaxation rate (R_2) values for different concentrations of the MMP suspended in methanol with 0.5% agarose. Based on these values, we calculated transverse relaxivity R_2=23.81±2.37 (mg/ml^{-1}s^{-1}).

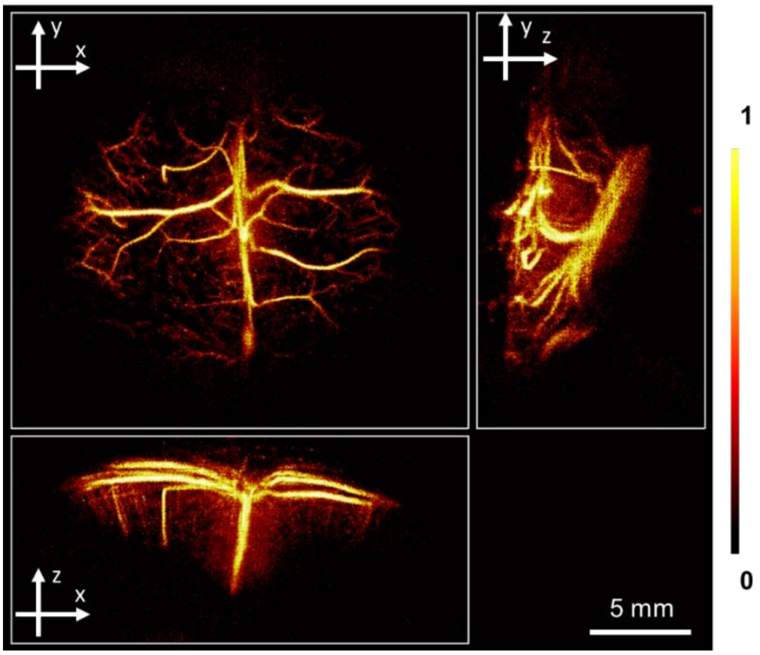

Figure S16: Maximum intensity projections of representative volumetric LOA images recorded non-invasively from a mouse brain. The displayed images were filtered using a Gaussian filter and had their dynamic range compressed using a logarithmic function followed by a sigmoid transformation[41].

	Actuation	Suitable types	Suitable scenarios	PROS	CONS
**Remote**	Magnetic	Magnetic	Deep tissue	• Noncontact actuation<br>• High penetration of tissue and body, and good biocompatibility<br>• Multiple motion modes<br>• High-precision manipulation	• Required complex equipment
	Light	Light	Superficial tissue	• Noncontact actuation<br>• Adjustable radiation energy<br>• Highly spatially selective	• Limited penetration
	Ultrasonic	Ultrasound	Deep tissue<br>Viscous solution	• Noncontact actuation<br>• High penetration of tissue and body and biocompatibility in the MHz range.<br>• A great degree of tunability	• Difficult to control precisely<br>• Affected by the environment
	Electric	Electrically conductive or electrical stimulus responsive	Ionic media (such as the interstitial fluid and blood)	• Contact-free and inexpensive<br>• Easy to modulate the electrical signal	• Short locomotion range<br>• Incompatible with highly ionic media such as tissue fluid and blood
**Local**	Chemical	chemical activity	Chemical fuel (such as H_2O_2, gastric juice)	• Energy-efficient and autonomous motion<br>• Dynamic in situ chemical processes	• Difficult to control the microrobot's speed, direction<br>• Requirements for the surrounding chemical environment.<br>• Limited actuation lifetimes

Table S1: Actuation Methods for mobile nanoparticles as micro/nanorobots

Concentration (mg/ml)	R_2 (1/s)
0.5	22.91161
0.4	19.80237
0.3	16.53466
0.2	14.10119
0.1	11.96387
0	11.43628

Table S2: Transverse relaxation rate (R_2) values for different concentrations of MMP

Study objective	**Frequency (kHz)**	**Maximum magnetic field (kA/m)**	**Duration (min)**	**Reference**
In vitro	118	30.6	30	42
Rats	150	11	20	43
Rats	100	18	40	44
Rats	118	30.6	30	45
Clinical trial	100	18	60	46
Clinical trial	100	15	60	47
This work	338	34.6	10	-

Table S3: Comparison of the RF fields applied in vivo in the literature

Supplementary Movie Legends

Movie S1- MMP’s response to the pattern magnetic field.

Movie S2- MMP’s rolling behavior on the glass plane.

Movie S3- MMP’s vortexing on the glass plane

Movie S4- Reconfigurable swarm behavior of MMP in the Helmholtz coils systems.

Movie S5- MMP’s upstream actuation against flowing fluid.

Movie S6- MMP’s dispersing behaviors in the tube.

Movie S7- MMP’s directional actuation in the channel of the Y-structure tube.

Movie S8- OA imaging of the MMP’s directional actuation in the tube.

Movie S9- OA imaging of the MMP’s locomotion in the pig brain surface vessel.

Movie S10- Visualization of the flow of individual MMP (maximum intensity projections) downstream and upstream behavior in the tail. Differential optoacoustic (OA) image (green colormap) is superimposed on the OA image (orange colormap).

Movie S11- Comparison of optoacoustic tomography (OAT) and localization optoacoustic tomography (LOT) at the brain. Rotating views are shown by visualization of the flow of individual MMP (maximum intensity projections). The differential optoacoustic (OA) image (green colormap) is superimposed on the OA image (orange colormap).

References

1. Bozuyuk, U. et al. Light-Triggered Drug Release from 3D-Printed Magnetic Chitosan Microswimmers. ACS Nano 12, 9617–9625 (2018).
2. Zheng, Z. et al. Ionic shape-morphing microrobotic end-effectors for environmentally adaptive targeting, releasing, and sampling. Nat Commun 12, 411 (2021).
3. Sridhar, V. et al. Light-driven carbon nitride microswimmers with propulsion in biological and ionic media and responsive on-demand drug delivery. Science Robotics 7, eabm1421(2022).
4. Sridhar, V. et al. Designing Covalent Organic Framework-Based Light-Driven Microswimmers toward Therapeutic Applications. Advanced Materials 35, 2301126 (2023).
5. Kaynak, M., Dirix, P. & Sakar, M. S. Addressable Acoustic Actuation of 3D Printed Soft Robotic Microsystems. Advanced Science 7, 2001120 (2020).
6. Hines, L., Petersen, K., Lum, G. Z. & Sitti, M. Soft Actuators for Small-Scale Robotics. Advanced Materials 29, 1603483 (2017).
7. Zhang, X. et al. Chitosan enhanced the stability and antibiofilm activity of self-propelled Prussian blue micromotor. Carbohydrate Polymers 299, 120134 (2023).
8. He, L. et al. Highly bioactive zeolitic imidazolate framework-8–capped nanotherapeutics for efficient reversal of reperfusion-induced injury in ischemic stroke. Science Advances 6, eaay9751 (2020).
9. Kaiser, A., Snezhko, A. & Aranson, I. S. Flocking ferromagnetic colloids. Science Advances 3, e1601469 (2017).
10. Wrede, P. et al. Real-time 3D optoacoustic tracking of cell-sized magnetic microrobots circulating in the mouse brain vasculature. Science Advances 8, eabm9132 (2022).
11. Liu, D., Guo, R., Wang, B., Hu, J. & Lu, Y. Magnetic Micro/Nanorobots: A New Age in Biomedicines. Advanced Intelligent Systems 4, 2200208 (2022).
12. Bozuyuk, U. et al. High-Performance Magnetic FePt (L10) Surface Microrollers Towards Medical Imaging-Guided Endovascular Delivery Applications. Advanced Functional Materials 32, 2109741 (2022).
13. Zhang, S. et al. 3D-printed micrometer-scale wireless magnetic cilia with metachronal programmability. Science Advances 9, eadf9462 (2023).
14. Lee, Y.-W. et al. Multifunctional 3D-Printed Pollen Grain-Inspired Hydrogel Microrobots for On-Demand Anchoring and Cargo Delivery. Advanced Materials 35, 2209812 (2023).
15. Giltinan, J., Sridhar, V., Bozuyuk, U., Sheehan, D. & Sitti, M. 3D Microprinting of Iron Platinum Nanoparticle-Based Magnetic Mobile Microrobots. Advanced Intelligent Systems 3, 2000204 (2021).
16. Lin, M. et al. A Combination Therapy of pHRE-Egr1-HSV-TK/Anti-CD133McAb-131I/MFH Mediated by FePt Nanoparticles for Liver Cancer Stem Cells. Journal of Nanomaterials 2020, e7180613 (2020).
17. Wang, Q. et al. Ultrasound Doppler-guided real-time navigation of a magnetic microswarm for active endovascular delivery. Science Advances 7, eabe5914 (2021).
18. Xie, H. et al. Reconfigurable magnetic microrobot swarm: Multimode transformation,

locomotion, and manipulation. Science Robotics 4, eaav8006 (2019).

19. Yang, M. et al. Swarming magnetic nanorobots bio-interfaced by heparinoid-polymer brushes for in vivo safe synergistic thrombolysis. Science Advances 9, eadk7251 (2023).
20. Zeyni, V., Karimi, S. & Namazi, H. Surface PEGylation of ZIF-8 metal-organic framework based on magnetic hydroxyapatite as a pH/magnetic targeting responsive system for anticancer drug delivery. Microporous and Mesoporous Materials 354, 112544 (2023).
21. Feng, S., Zhang, X., Shi, D. & Wang, Z. Zeolitic imidazolate framework-8 (ZIF-8) for drug delivery: A critical review. Front. Chem. Sci. Eng. 15, 221–237 (2021).
22. M. Sreekanth, T. V., Prasad, K., Yoo, J., Kim, J. & Yoo, K. ZIF-8@CoFe 2 O 4 as a highly efficient bifunctional electrocatalyst for the methanol oxidation and oxygen evolution reactions. Catalysis Science & Technology 13, 3445–3455 (2023).
23. Song, Y. et al. Biodegradable Imprinted Polymer Based on ZIF-8/DOX-HA for Synergistically Targeting Prostate Cancer Cells and Controlled Drug Release with Multiple Responses. ACS Appl. Mater. Interfaces 15, 25339–25353 (2023).
24. Guo, H., Liu, L., Hu, Q. & Dou, H. Mixed Solvent Method for Improving the Size Uniformity and Cargo-Loading Efficiency of ZIF-8 Nanoparticles. Langmuir 37, 10089–10099 (2021).
25. Tran, V. A. & Lee, S.-W. pH-triggered degradation and release of doxorubicin from zeolitic imidazolate framework-8 (ZIF8) decorated with polyacrylic acid. RSC Adv. 11, 9222–9234 (2021).
26. M. El-Metwaly, N., A. Katouah, H., El-Desouky, M. G., El-Bindary, A. A. & El-Bindary, M. A. Fabricating of Fe3O4@Ag-MOF nanocomposite and evaluating its adsorption activity for removal of doxorubicin. Journal of Environmental Science and Health, Part A 57, 1099–1115 (2022).
27. Zeyni, V., Karimi, S. & Namazi, H. Surface PEGylation of ZIF-8 metal-organic framework based on magnetic hydroxyapatite as a pH/magnetic targeting responsive system for anticancer drug delivery. Microporous and Mesoporous Materials 354, 112544 (2023).
28. Nanosized metal–organic frameworks as unique platforms for bioapplications. Chemical Communications 59, 2869–2887 (2023).
29. Wrede, P. et al. Real-time 3D optoacoustic tracking of cell-sized magnetic microrobots circulating in the mouse brain vasculature. Science Advances 8, eabm9132 (2022).
30. Wang, Q. et al. Tracking and navigation of a microswarm under laser speckle contrast imaging for targeted delivery. Science Robotics 9, eadh1978 (2024).
31. Wu, Z. et al. A microrobotic system guided by photoacoustic computed tomography for targeted navigation in intestines in vivo. Science Robotics 4, eaax0613 (2019).
32. Ntziachristos, V. Going deeper than microscopy: the optical imaging frontier in biology. Nat Methods 7, 603–614 (2010).
33. Razansky, D. et al. Multispectral opto-acoustic tomography of deep-seated fluorescent proteins in vivo. Nature Photon 3, 412–417 (2009).
34. L. Deán-Ben, X., Gottschalk, S., Larney, B. M., Shoham, S. & Razansky, D. Advanced optoacoustic methods for multiscale imaging of in vivo dynamics. Chemical Society Reviews

46, 2158–2198 (2017).

35. Wang, L. V. & Hu, S. Photoacoustic Tomography: In Vivo Imaging from Organelles to Organs. Science 335, 1458–1462 (2012).
36. Yao, J. et al. Multiscale photoacoustic tomography using reversibly switchable bacterial phytochrome as a near-infrared photochromic probe. Nat Methods 13, 67–73 (2016).
37. Dutz, S. & Hergt, R. Magnetic particle hyperthermia—a promising tumour therapy? Nanotechnology 25, 452001 (2014).
38. Sebastian, A. R., Kim, H. J. & Kim, S. H. Analysis and control of heating of magnetic nanoparticles by adding a static magnetic field to an alternating magnetic field. Journal of Applied Physics 126, 134902 (2019).
39. Carrey, J., Mehdaoui, B. & Respaud, M. Simple models for dynamic hysteresis loop calculations of magnetic single-domain nanoparticles: Application to magnetic hyperthermia optimization. Journal of Applied Physics 109, 083921 (2011).
40. Zheng, D., Ramos-Sebastian, A., Jung, W. S. & Kim, S. H. Fabrication and preliminary evaluation of alginate hydrogel-based magnetic springs with actively targeted heating and drug release mechanisms for cancer therapy. Composites Part B: Engineering 230, 109551 (2022).
41. L. Deán-Ben, X., Gottschalk, S., Larney, B. M., Shoham, S. & Razansky, D. Advanced optoacoustic methods for multiscale imaging of in vivo dynamics. Chemical Society Reviews 46, 2158–2198 (2017).
42. Shinkai, M. et al. Intracellular hyperthermia for cancer using magnetite cationic liposomes: in vitro study. Jpn J Cancer Res 87, 1179–1183 (1996).
43. I. Rabias, D. Tsitrouli, E. Karakosta, T. Kehagias, G. Diamantopoulos, M. Fardis, D. Stamopoulos, T. G. Maris, P. Falaras, N. Zouridakis, N. Diamantis, G. Panayotou, D. A. Verganelakis, G. I. Drossopoulou, E. C. Tsilibari, G. Papavassiliou, Rapid magnetic heating treatment by highly charged maghemite nanoparticles on Wistar rats exocranial glioma tumors at microliter volume. Biomicrofluidics. 4, 1–8 (2010).
44. A. Jordan, R. Scholz, K. Maier-Hauff, F. K. H. van Landeghem, N. Waldoefner, U. Teichgraeber, J. Pinkernelle, H. Bruhn, F. Neumann, B. Thiesen, A. von Deimling, R. Felix, The effect of thermotherapy using magnetic nanoparticles on rat malignant glioma. J. Neurooncol. 78, 7–14 (2006).
45. M. Yanase, M. Shinkai, H. Honda, T. Wakabayashi, J. Yoshida, T. Kobayashi, Antitumor Immunity Induction by Intracellular Hyperthermia Using Magnetite Cationic Liposomes. Japanese J. Cancer Res. 89, 775–782 (1998).
46. K. Maier-Hauff, R. Rothe, R. Scholz, U. Gneveckow, P. Wust, B. Thiesen, A. Feussner, A. Deimling, N. Waldoefner, R. Felix, A. Jordan, Intracranial thermotherapy using magnetic nanoparticles combined with external beam radiotherapy: Results of a feasibility study on patients with glioblastoma multiforme. J. Neurooncol. 81, 53–60 (2007).
47. K. Maier-Hauff, F. Ulrich, D. Nestler, H. Niehoff, P. Wust, B. Thiesen, H. Orawa, V. Budach, A. Jordan, Efficacy and safety of intratumoral thermotherapy using magnetic iron-oxide

nanoparticles combined with external beam radiotherapy on patients with recurrent glioblastoma multiforme. J. Neurooncol. 103, 317–324 (2011).
